# Disease-associated KBTBD4 mutations in medulloblastoma elicit neomorphic ubiquitylation activity to promote CoREST degradation

**DOI:** 10.1038/s41418-022-00983-4

**Published:** 2022-04-04

**Authors:** Zhuoyao Chen, Rafael M. Ioris, Stacey Richardson, Ava N. Van Ess, Iolanda Vendrell, Benedikt M. Kessler, Francesca M. Buffa, Luca Busino, Steven C. Clifford, Alex N. Bullock, Vincenzo D’Angiolella

**Affiliations:** 1 Centre for Medicines Discovery, Nuffield Department of Medicine, University of Oxford, Oxford, OX3 7DQ UK; 2 Cancer Research UK and Medical Research Council Oxford Institute for Radiation Oncology, Department of Oncology, University of Oxford, Oxford, OX3 7DQ UK; 3 Wolfson Childhood Cancer Research Centre, Newcastle University Centre for Cancer, Newcastle upon Tyne, NE1 7RU UK; 4 Target Discovery Institute, Nuffield Department of Medicine, University of Oxford, Oxford, OX3 7FZ UK; 5 Department of Cancer Biology and Abramson Family Cancer Research Institute, Perelman School of Medicine, University of Pennsylvania, Philadelphia, PA 19104 USA

**Keywords:** Ubiquitin ligases, CNS cancer

## Abstract

Medulloblastoma is the most common malignant brain tumour in children. Genomic studies have identified distinct disease subgroups: wnt/wingless (WNT), sonic hedgehog (SHH), and non-WNT/non-SHH, comprising group 3 and group 4. Alterations in WNT and SHH signalling form the pathogenetic basis for their subgroups, whereas those for non-WNT/non-SHH tumours remain largely elusive. Recent analyses have revealed recurrent in-frame insertions in the E3 ubiquitin ligase adaptor Kelch Repeat and BTB Domain Containing 4 (KBTBD4) in cases of group 3/4 medulloblastoma. Critically, group 3/4 tumours with KBTBD4 mutations typically lack other gene-specific alterations, such as *MYC* amplification, indicating KBTBD4 insertion mutations as the primary genetic driver. Delineating the role of KBTBD4 mutations thus offers significant opportunities to understand tumour pathogenesis and to exploit the underpinning mechanisms therapeutically. Here, we show a novel mechanism in cancer pathogenesis whereby indel mutations in KBTBD4 drive its recognition of neo-substrates for degradation. We observe that KBTBD4 mutants promote the recruitment and ubiquitylation of the REST Corepressor (CoREST), which forms a complex to modulate chromatin accessibility and transcriptional programmes. The degradation of CoREST promoted by KBTBD4 mutation diverts epigenetic programmes inducing significant alterations in transcription to promote increased stemness of cancer cells. Transcriptional analysis of >200 human group 3 and 4 medulloblastomas by RNA-seq, highlights the presence of CoREST and stem-like signatures in tumours with KBTBD4 mutations, which extend to a further sub-set of non-mutant tumours, suggesting CoREST alterations as a novel pathogenetic mechanism of wide relevance in groups 3 and 4. Our findings uncover KBTBD4 mutation as a novel driver of epigenetic reprogramming in non-WNT/non-SHH medulloblastoma, establish a novel mode of tumorigenesis through gain-of-function mutations in ubiquitin ligases (neo-substrate recruitment) and identify both mutant KBTBD4 and CoREST complexes as new druggable targets for improved tumour-specific therapies.

## Introduction

Cullin-RING Ligases (CRLs) represent a class of multi-subunit E3s, in which the cullin subunit forms a central scaffold to recruit interchangeable adaptors for substrate recruitment and ubiquitin-charged E2s for substrate ubiquitylation. The prototypical CRL1s assemble with Cul1, Skp1 and a variable substrate recruitment subunit called an F-box protein to form a Skp1-Cul1-F-box (SCF) ubiquitin ligase [1–3]. In SHH medulloblastoma, the substrate adaptor F-box/LRR-repeat protein 17 (Fbxl17) assembles into a functional SCF^Fbxl17^ to promote the ubiquitylation-dependent degradation of the tumour suppressor Suppressor of fused homologue (SUFU), acting as a bona-fide oncoprotein [4]. However, the function of other CRL adaptors in medulloblastoma is largely unexplored.

Recurrent heterozygous in-frame insertion mutations in the Cullin RING ubiquitin Ligase 3 (CRL3) adaptor KBTBD4 are reported in children with non-WNT/non-SHH medulloblastoma [5], as well as in children and adults with Pineal Parenchymal Tumours of Intermediate Differentiation (PPTID) [6]. Recent analyses also reveal recurrent in-frame insertions in KBTBD4 in 40% of cases of adult group 3 medulloblastomas [7].

**Fig. 1:**
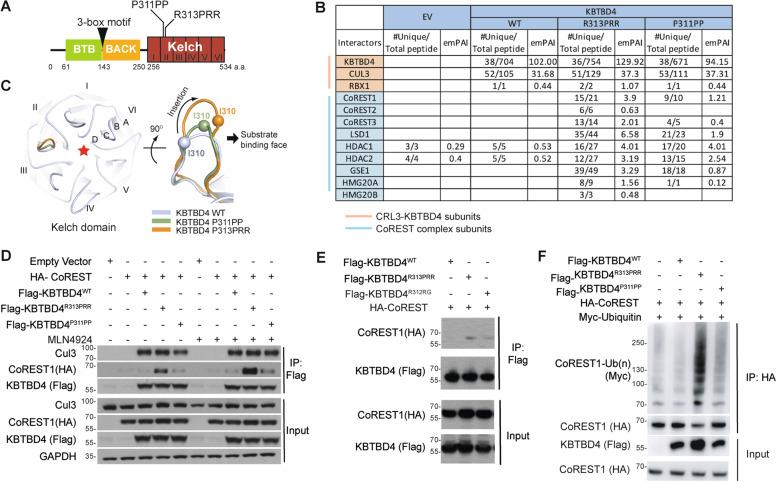
KBTBD4 mutations elicit neomorphic activity promoting the recognition and ubiquitylation of CoREST. **A** Domain organisation of KBTBD4. The Kelch domain contains 6 Kelch repeat motifs (I-VI) that form the six blades of a β-propeller fold. Recurrent indel mutations KBTBD4^R313PRR^ and KBTBD4^P311PP^ map to the second Kelch repeat. **B** HEK293T cells transfected with empty vector (EV), Flag-KBTBD4^WT^, Flag-KBTBD4^R313PRR^ or Flag-KBTBD4^P311PP^ were immunoprecipitated and eluted with Flag-peptide. Eluates were digested and analysed by LC-MS/MS. Cells were treated with 100 nM MLN4924 for 4 h before harvesting. The table highlights interactors from the CRL3^KBTBD4^ and CoREST complexes (red and light blue shading, respectively). **C** Structural model of the KBTBD4 Kelch domain (left). Red star indicates the substrate-binding pocket. Indel mutations are predicted to lengthen the substrate-binding loop in blade II to support neo-substrate interaction (right). The inserted mutant amino acids (denoted by an arrow) lie on the outer edge of the loop that faces away from the central substrate pocket. N-terminal wild-type residues preceding the mutation site are therefore repositioned into the substrate pocket (Ile310 Cα atoms in each model are shown as spheres for reference). **D** HEK293T cells were co-transfected with HA-tagged CoREST and an Empty Vector, Flag-KBTBD4^WT^, Flag-KBTBD4^R313PRR^ or Flag-KBTBD4^R311PP^ and were treated with MLN4924. Whole-cell extracts were immunoprecipitated (IP) with anti-FLAG resin, and immunoprecipitates were immunoblotted as indicated. **E** HEK293T cells were co-transfected with HA-CoREST along with Flag‐KBTBD4^WT^, Flag-KBTBD4^R313PRR^ or Flag-KBTBD4^R312RG^ as indicated (+). After treatment with MLN4924 cell lysates were immunoprecipitated by anti‐FLAG resin and immunoblotted as indicated. **F** HEK293T cells were co-transfected with MYC‐tagged ubiquitin and HA-CoREST along with Flag‐KBTBD4^WT^, Flag-KBTBD4^R313PRR^ or Flag-KBTBD4^P311PP^ as indicated (+). HA‐CoREST was immunoprecipitated with anti‐HA resin under denaturing conditions and immunoblotted. Cells were treated with 5 μM MG132 for 4 h before harvesting. The poly-ubiquitylated CoREST (indicated by brackets) was detected by immunoblot with anti-Myc antibody.

KBTBD4 forms a multi-subunit E3 using a BTB domain to engage Cul3 and a Kelch domain to recruit substrates. KBTBD4 mutations recur around the same conserved residues (G308 to R313) in the Kelch domain (Fig. 1A and Supplementary Fig. S1). CRL3 adaptors have critical roles in cancer pathogenesis and hotspot inactivating mutations have been identified in the substrate recruitment domain of Kelch-like protein 6 (KLHL6), favouring the growth of diffuse large B-cell lymphoma [8].

Here, we present the first prototypical example of neomorphic mutation in an E3 ubiquitin ligase. We show that medulloblastoma-associated hotspot mutations in the Kelch domain of KBTBD4 promote the recruitment of CoREST as a neo-substrate for ubiquitylation and degradation. CoREST1/2/3 form critical scaffolds for chromatin modifying complexes, which incorporate lysine-specific histone demethylase 1A (LSD1) and histone deacetylase 1/2 (HDAC1/2) for the demethylation and deacetylation of histones at H3K4 in vitro and in vivo [9, 10]. We observe that degradation of CoREST by mutant KBTBD4 leads to increases in histone H3 methylation at lysine 4 (H3K4 mono- and di-methylation) and altered transcription. We further show that mutant KBTBD4 activates the transcription of CoREST targets favouring stemness, known to promote tumorigenesis in medulloblastoma [11].

## Results

### Medulloblastoma-associated KBTBD4 mutations drive recognition of the CoREST complex

As an initial strategy to identify substrates of KBTBD4, we immunoprecipitated KBTBD4^WT^, KBTBD4^R313PRR^ and KBTBD4^P311PP^ (the most recurrent mutations in group 3 and 4 medulloblastomas, respectively) (Fig. 1A) [5] from HEK293T cells and performed tryptic digestion and Liquid Chromatography/Tandem Mass Spectrometry (LC-MS/MS) to analyse their interacting partners. We identified specific components of the CRL3 machinery in KBTBD4^WT^, KBTBD4^R313PRR^ and KBTBD4^P311PP^ immunoprecipitates, including Cul3 and Rbx1. Strikingly, we could detect CoREST complex subunits only in KBTBD4^R313PRR^ and KBTBD4^P311PP^ immunoprecipitates, but not in that of KBTBD4^WT^, suggesting a putative mutant-specific neofunction (Fig. 1B). A Venn diagram comparing differential interacting partners of KBTBD4^WT^, KBTBD4^R313PRR^ and KBTBD4^P311PP^ identified in LC-MS/MS is presented in Supplementary Fig. S2A.

While the mutations in KBTBD4 are different in terms of amino acid composition and insertion length, the same CoREST complex was identified in the LC-MS/MS of different mutants, including CoREST, LSD1, HDAC1/2, Genetic Suppressor Element 1 (GSE1) and High Mobility Group 20A (HMG20A/B) (Fig. 1B). Using known Kelch domain structures [12–14], we mapped the 6 Kelch repeats in KBTBD4 and constructed 3D structural models for KBTBD4^WT^ and oncogenic mutants. The recurrent indel mutations KBTBD4^R313PRR^ and KBTBD4^P311PP^ map to the same Kelch domain loop region for substrate recruitment (Fig. 1A). Here, the in-frame insertions are predicted to lengthen the substrate-binding loop to support neo-substrate interaction (Fig. 1C). Importantly, the inserted mutant amino acids lie on the outer edge of the loop that faces away from the central substrate pocket. It is therefore the N-terminal wild-type residues preceding the mutation site that are predicted to shift position to engage the CoREST substrates. This structural model therefore presents a possible explanation for how different in-frame insertions can modify the substrate-binding surface to promote similar neo-substrate recruitment (Fig. 1C).

To confirm the LC-MS/MS data and our model of neo-substrate recruitment, we first tested the interaction between KBTBD4^WT^, KBTBD4^R313PRR^, KBTBD4^P311PP^ and CoREST by co-expressing these proteins in HEK293T cells. We could co-immunoprecipitate the CoREST complex with both KBTBD4^R313PRR^ and KBTBD4^P311PP^, but not KBTBD4^WT^ (Fig. 1D). This interaction was increased by treating the cells with MLN4924, which inhibits the activity of Nedd8-Activating Enzyme (NAE1). Neddylation of Cullins is required for the activity of CRL complexes; MLN4924 therefore blocks all CRL activity in cells and stabilises CRL-bound substrates [15]. Thus, the increased interaction between mutant KBTBD4 and CoREST in the presence of MLN4924 reflects the fact that KBTBD4^R313PRR^ and KBTBD4^P311PP^ can interact with CoREST, but not ubiquitylate or degrade it due to the inactivation of CRL3^KBTBD4^ by MLN4924 (Fig. 1D). Of note, KBTBD4^WT^, KBTBD4^R313PRR^ and KBTBD4^P311PP^ all bound equivalently to Cul3, showing that changes in CRL3 assembly were not related to the substrate specificity of KBTBD4 variants (Fig. 1D). Interaction of exogenously expressed CoREST was also observed with KBTBD4^R313PRR^ and KBTBD4^P311PP^ but not KBTBD4^WT^ after immunoprecipitation of CoREST (Supplementary Fig. S2B).

To further validate our model, we tested the interaction of another tumorigenic mutant of KBTBD4, namely KBTBD4^R312RG^. Again we detected interaction of KBTBD4^R312RG^ with CoREST at comparable levels to the interaction detected with KBTBD4^R313PRR^ (Fig. 1E). To confirm the formation of functional CRL3^KBTBD4^ directly, and to test whether CoREST is a genuine substrate, we measured the ubiquitylation activity of CRL3^KBTBD4^ on CoREST. Compared to empty vector background, both KBTBD4^R313PRR^ and KBTBD4^P311PP^ promoted ubiquitylation of CoREST, whereas KBTBD4^WT^ did not, in accordance with the interaction data (Fig. 1F). The CoREST interaction with KBTBD4^P311PP^ appeared weaker than with KBTBD4^R313PRR^, and the ubiquitylation levels of CoREST were consistent with the extent of interaction (Fig. 1D, F). We also observed that CoREST ubiquitylation was prevented by MLN4924 (Supplementary Fig. S2C) as predicted from the results of immunoprecipitation analysis (Fig. 1D).

### Expression of KBTBD4 mutants in medulloblastoma cancer cells induces CoREST degradation and epigenetic rewiring

The experiments presented above used HEK293T cells. We subsequently derived several different medulloblastoma cell lines stably expressing KBTBD4^WT^, KBTBD4^R313PRR^ and KBTBD4^P311PP^ to confirm our findings in more physiologically relevant models. In DAOY medulloblastoma cell lines, we could detect interaction of endogenous CoREST with KBTBD4^P311PP^, but not with KBTBD4^WT^ as in HEK293T (Supplementary Fig. S3A). Expression of mutant KBTBD4^R313PRR^ or KBTBD4^P311PP^, but not KBTBD4^WT^, promoted a drastic reduction in CoREST levels, which could be restored by treating cells with the proteasome inhibitor MG132 (Supplementary Fig. S3B), showing that CoREST is targeted for proteasomal degradation by KBTBD4 mutants, but not KBTBD4^WT^, in accordance with the interaction data. MLN4924 similarly prevented the degradation of CoREST in DAOY cell lines expressing KBTBD4^R313PRR^ or KBTBD4^P311PP^, demonstrating a role for CRL activity in controlling the levels of CoREST (Supplementary Fig. S3C).

While DAOY cells are the most cited medulloblastoma cell-based model, they do not represent the non-WNT/non-SHH medulloblastoma classes in which the KBTBD4 mutations are detected. We therefore sought to confirm our observations in other more representative cells. Medulloblastoma cell lines derived in the past 4 decades (*n* > 40), collectively show a surprising lack of genetic diversity and medulloblastoma cell lines containing mutant KBTBD4 have not been reported to date [16]. To establish suitable models to investigate KBTBD4, we assessed endogenous CoREST and LSD1 protein levels in five representative medulloblastoma cell lines of different subgroups: D283Med (group 3/4, without reported *MYC* amplification), HD-MB03 (group 3, *MYC* amplification), D425Med (group 3, *MYC* amplification), D458Med (metastatic counterpart of D425Med primary tumour) as well as DAOY (SHH subgroup). All lines showed detectable CoREST and LSD1 proteins (Supplementary Fig. S3D). We used lentiviral expression to stably introduce KBTBD4^WT^, KBTBD4^R313PRR^ or KBTBD4^P311PP^ in the group 3 medulloblastoma cell line D425Med. Cells expressing mutant KBTBD4 showed a reduction in CoREST protein level, whereas these levels were unchanged by expression of KBTBD4^WT^ (Supplementary Fig. S3E), consistent with data from other medulloblastoma cell lines.

**Fig. 2:**
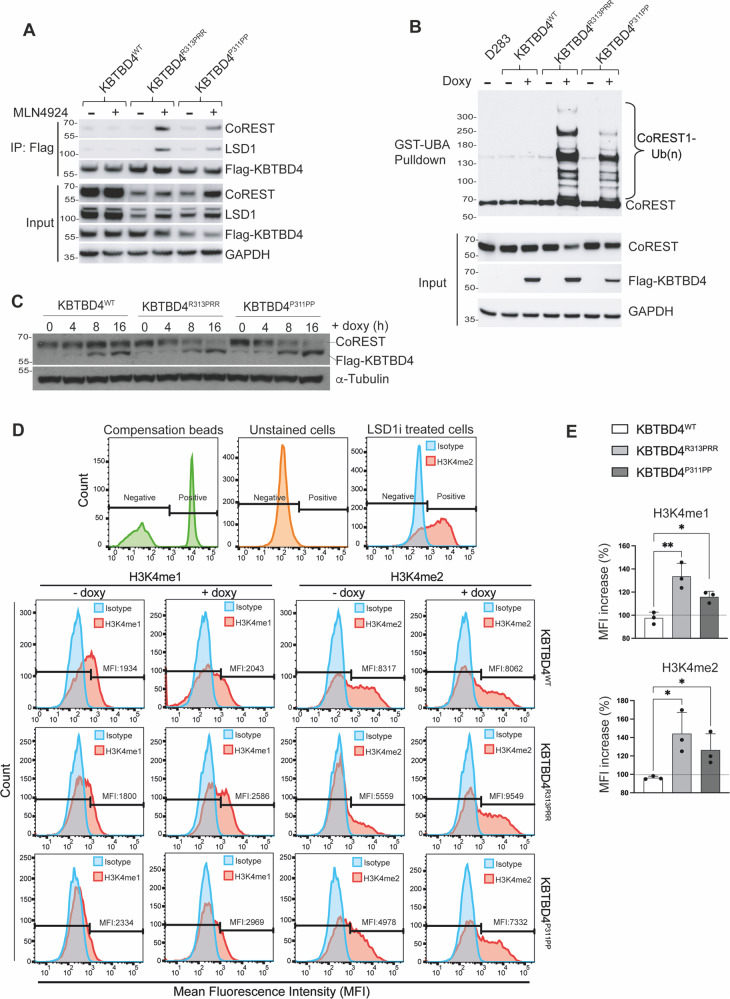
Expression of KBTBD4 mutants in medulloblastoma cells promotes CoREST ubiquitylation and modifies epigenetic marks. **A** D283Med cells expressing Flag‐KBTBD4^WT^, Flag-KBTBD4^R313PRR^ or Flag-KBTBD4^P311PP^ after doxycycline induction (1 µg/mL for 40 h) were treated with 100 nM MLN4924 for 4 h as indicated (+). After lysis, extracts were immunoprecipitated with Flag-resin and immunoblotted as indicated. **B** D283Med cells expressing Flag‐KBTBD4^WT^, Flag-KBTBD4^R313PRR^ or Flag-KBTBD4^P311PP^ were treated with doxycycline (1 µg/mL) for 16 h and lysed. Endogenous ubiquitylated proteins were isolated using GST fused ubiquitin binding domain UBA^Ubq^ and separated by SDS-PAGE. Isolated proteins were detected by immunoblot as indicated. All samples were treated with MG132 for 6 h. **C** D283Med cells containing Flag‐KBTBD4^WT^, Flag-KBTBD4^R313PRR^ or Flag-KBTBD4^P311PP^ were treated with doxycycline (1 µg/mL) for the indicated time (h = hours). Samples were separated by SDS-page and immunoblotted as indicated. **D**, **E** Flow cytometry analysis. **D** (*Top three panels*) Representative images showing the gating procedure where Negative and Positive gates were determined using 5 controls: Compensation beads (green), unstained cells (orange), cells stained only with secondary antibody (not shown), cells treated with LSD1 inhibitor followed by incubation with either isotype (Blue) or H3K4me2 (red) primary antibodies and secondary antibody. The remaining panels are representative images showing the results related to flow cytometry analysis of H3K4me1 and H3K4me2 in D283Med cells expressing Flag‐KBTBD4^WT^, Flag-KBTBD4^R313PRR^ or Flag-KBTBD4^P311PP^ after doxycycline induction (1 µg/mL for 4 days). **E** Bar charts showing the quantification of the analysis in **D**, plotted as percentage increase of the Mean Fluorescence Intensity (MFI) of positive cells where controls (- doxy) were set as 100% (dashed line). Data are presented as mean ±standard deviation (SD), with at least three independent experiments. *P* values (*P < 0.05 and **P < 0.005) were calculated by paired and two‐tailed *t*‐test.

We reasoned that the D283Med cell line represented a particularly suitable model to investigate the role of KBTBD4 mutations in medulloblastoma as, in terms of transcriptional identity, D283Med are close to the identified subtype II of a more recent medulloblastoma classification of group 3 and 4 subtypes [17]. We created D283Med and D425Med lines in which the expression of KBTBD4^WT^, KBTBD4^R313PRR^ and KBTBD4^P311PP^ was controlled by addition of doxycycline. After induction of KBTBD4^WT^, KBTBD4^R313PRR^ or KBTBD4^P311PP^ expression in D283Med, we measured their respective interaction with endogenous CoREST complex. We detected interaction of CoREST and LSD1 with KBTBD4^R313PRR^ and KBTBD4^P311PP^ exclusively in cells treated with MLN4924 where the ubiquitylation activity of CRL3 was prevented (Fig. 2A).

To assess the capacity of KBTBD4^WT^, KBTBD4^R313PRR^ and KBTBD4^P311PP^ to ubiquitylate endogenous CoREST in the absence of overexpressed ubiquitin, we isolated endogenous ubiquitylated proteins using a GST fused ubiquitin binding domain UBA^Ubq^ previously reported [18] and detected the ubiquitylation formed on endogenous CoREST. KBTBD4^R313PRR^ and KBTBD4^P311PP^ promoted ubiquitylation of endogenous CoREST, while ubiquitylation of CoREST in parental D283Med cells or in the presence of KBTBD4^WT^ was undetectable (Fig. 2B).

To determine the fate of ubiquitylated CoREST, we measured CoREST levels after addition of doxycycline. At comparable expression levels of KBTBD4^WT^, KBTBD4^R313PRR^ and KBTBD4^P311PP^ we observed a reduction in the levels of CoREST only upon expression of KBTBD4 mutants, consistent with CoREST degradation (Fig. 2C). Despite similar or higher levels of expression, KBTBD4^WT^ did not affect CoREST levels. To exclude that the effect observed on CoREST was due to overexpression artefacts of KBTBD4, we tested the levels of CoREST after inducing KBTBD4^WT^ and KBTBD4^P311PP^ with increasing concentrations of doxycycline. Minimal induction of KBTBD4^P311PP^ with the lowest doxycycline dose still promoted CoREST degradation (Supplementary Fig. S3F). By contrast, the highest expression of KBTBD4^WT^ did not alter CoREST levels (Supplementary Fig. S3F). In addition to CoREST, LSD1 was also degraded, although at a seemingly reduced level compared to CoREST (Supplementary Fig. S3F).

CoREST exerts its activity through LSD1, which is known to demethylate mono- and di-methylated H3K4 in vitro and in vivo [9, 10]. We assessed the impact of KBTBD4^R313PRR^ and KBTBD4^P311PP^ expression on the chromatin modifications mediated by CoREST and observed an increase of H3K4 mono- and di-methylation in accordance with loss of CoREST and LSD1 activity (Fig. 2D, E). The increase in the epigenetic mark H3K4me2 was also confirmed by Western blotting in D283Med and DAOY cell lines (Supplementary Fig. S3G, H). Overall, the data presented highlight that KBTBD4^R313PRR^ and KBTBD4^P311PP^ can promote the degradation of CoREST complex and increase H3K4 mono- and di-methyl epigenetic marks. The data also exclude that KBTBD4^WT^ targets CoREST under physiological conditions.

### Expression of KBTBD4 mutants in medulloblastoma cells promotes the de-repression of CoREST target genes activating pro-tumorigenic programmes

**Fig. 3:**
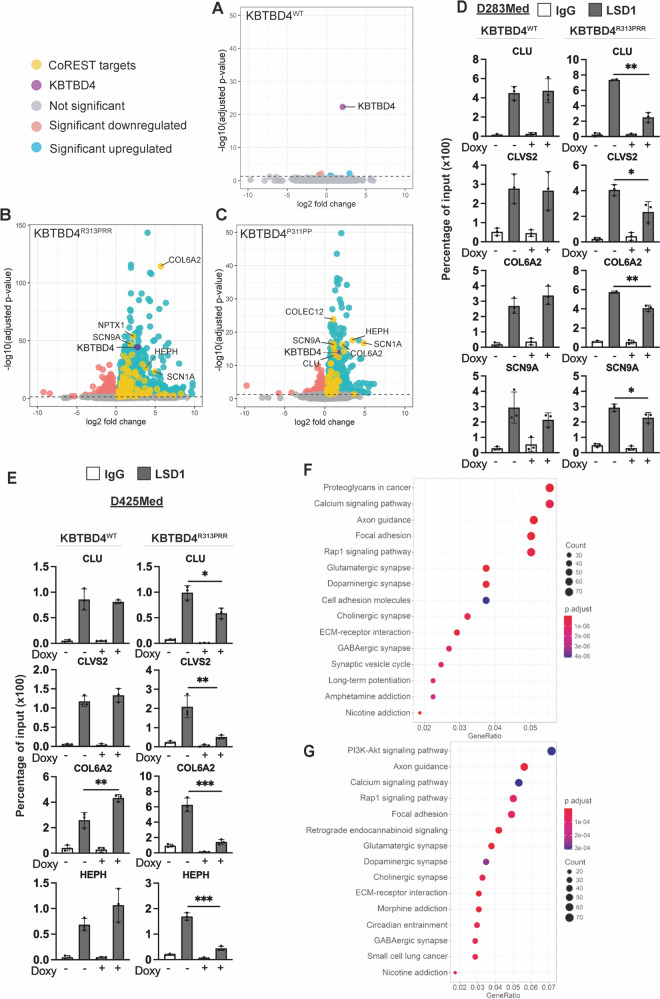
Expression of KBTBD4 mutants in medulloblastoma promotes the de-repression of CoREST target genes activating pro-tumorigenic programmes. Volcano plots showing the differential gene expression analysis from D283Med cells expressing (**A**) Flag‐KBTBD4^WT^, (**B**) Flag-KBTBD4^R313PRR^ or (**C**) Flag-KBTBD4^P311PP^ treated with doxycycline (1 µg/mL) for four days compared to their untreated controls. Coloured dots represent differentially expressed genes between two groups with *P*-adjust <0.05. KBTBD4 (indicated in Purple) and known CoREST target genes (yellow) are highlighted. ChIP analysis of LSD1 occupancy on gene promoters in (**D**) D283Med and (**E**) D425Med cells expressing Flag‐KBTBD4^WT^ or Flag-KBTBD4^R313PRR^ upon doxycycline (1 µg/mL) treatment for 3 days. Data are expressed as percentage of input. **F**, **G** Dotplot of KEGG pathway enrichment analysis of genes differentially upregulated comparing either KBTBD4^R313PRR^ or KBTBD4^P311PP^ treated with doxycycline versus untreated controls. The X axis shows the gene ratio which is the percentage of genes enriched in a KEGG term. The Y axis represents the enriched pathways; the size of the node represents the number of enriched genes within a particular term.

The impact of mutant KBTBD4 on epigenetic markers suggested the potential for altered transcription through de-repression of CoREST targets. To identify the transcriptional programmes promoted by mutant KBTBD4, we compared total RNA-seq samples in quadruplicates from D283Med cells where expression of KBTBD4^WT^, KBTBD4^R313PRR^ or KBTBD4^P311PP^ was promoted by doxycycline *versus* untreated controls (Supplementary Fig. S4A, B). Although we detected a significant induction of KBTBD4^WT^, a volcano plot and heatmap of differentially expressed genes revealed that KBTBD4^WT^ did not promote significant transcriptional changes (Fig. 3A and Supplementary Fig. S4B). By contrast, the expression of KBTBD4^R313PRR^ and KBTBD4^P311PP^ promoted increased expression of a specific genes, of which many were known CoREST target genes. Among 249 significantly upregulated genes, 130 genes in the KBTBD4^R313PRR^ sample and 77 in KBTBD4^P311PP^ were known CoREST targets (Fig. 3B, C and Supplementary Table S1). An increase in gene expression is expected following the loss of a transcriptional repressor like CoREST and establishes that mutant KBTBD4 alters transcription by controlling CoREST complex activity.

To confirm that the transcriptional alterations detected are consistent in different models we also used the D425Med cell line, where expression of KBTBD4^R313PRR^ or KBTBD4^P311PP^ faithfully leads to CoREST degradation (Supplementary Fig. S2E). Using quantitative PCR (qPCR) in cells expressing KBTBD4^R313PRR^ or KBTBD4^P311PP^ we could detect the upregulation of multiple CoREST targets (Supplementary Fig. S4C), confirming the role of mutant KBTBD4 in different model cell lines.

Furthermore, to establish a direct relationship between alterations in gene transcription and CoREST complex ubiquitylation by KBTBD4^R313PRR^ or KBTBD4^P311PP^, we performed Chromatin Immuno-Precipitation (ChIP) of LSD1 in D283Med and D425Med cell lines (Fig. 3D, E). Since LSD1 is also present in other chromatin remodelling complexes besides the ones formed by CoREST, we reasoned that, by using LSD1 ChIP, we could detect the recruitment and activity of the CoREST complex at the CoREST target genes in an unbiased way (i.e. independently of CoREST degradation). While in KBTBD4^WT^ expressing cells we did not detect a difference, both D425Med and D283Med lines expressing KBTBD4^R313PRR^ had significantly lower chromatin recruitment of LSD1 at four out of four CoREST target genes (Fig. 3D, E). Interestingly, the changes observed in gene expression after induction of KBTBD4 mutants could be in part recapitulated by siRNA mediated depletion of CoREST, reinforcing the idea that KBTBD4 mutants are mainly acting through CoREST depletion (Supplementary Fig. S3D).

To identify the transcriptional programmes contributing to tumorigenesis induced by KBTBD4^R313PRR^ and KBTBD4^P311PP^ expressing cells we also performed Kyoto Encyclopaedia of Genes and Genomes (KEGG) pathway analysis. We identified several cancer-related pathways (PI3K, RAP1 and proteoglycans in cancer) significantly upregulated in cells expressing KBTBD4 mutants (Fig. 3F, G) in accordance with pro-tumorigenic features. In addition, we detected a significant enrichment of pro-neurogenic programmes including axon guidance and glutamatergic synapses. While these programmes do not favour proliferation, they may be indicative of progenitor programmes specifying cellular identities favoured to grow in the cerebellum. Overall, the experiments presented above show that KBTBD4 mutants activate transcription by modulating CoREST complex occupancy and activity on chromatin, enabling the activation of specific cellular programmes for tumorigenesis and neurogenesis.

### KBTBD4 mutants enhance stemness of medulloblastoma cancer cells

**Fig. 4:**
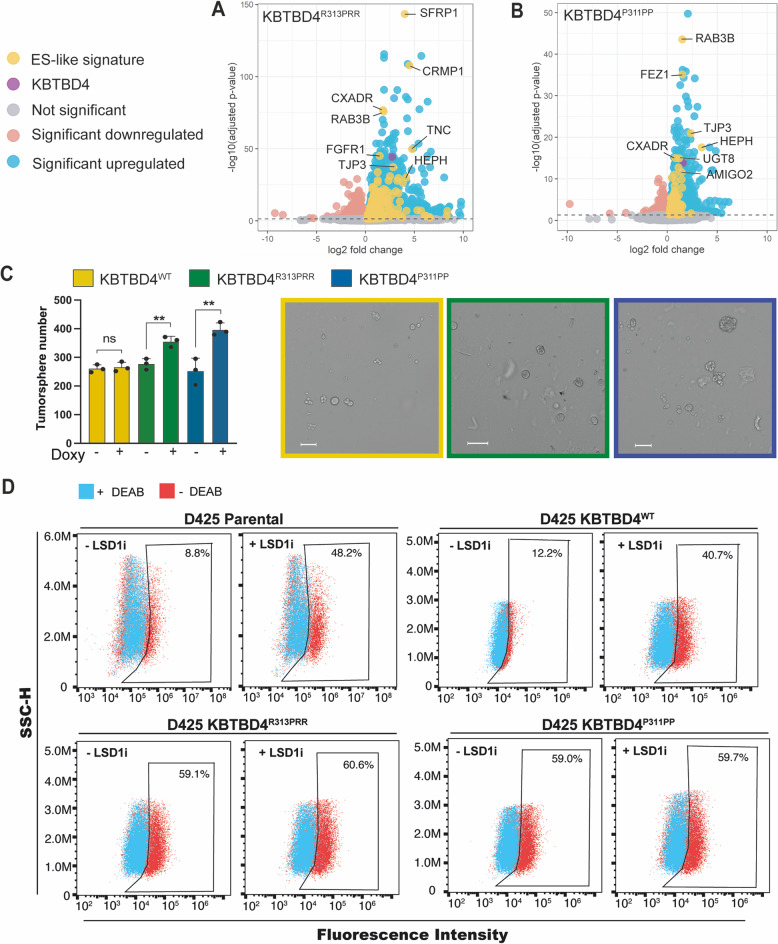
Mutant KBTBD4 enhances stemness of medulloblastoma cells. Volcano plot showing upregulated ES-like signature genes (yellow dots). RNA-seq upregulated genes (*P*-adjust <0.05) between (**A**) KBTBD4^R313PRR^ and (**B**) KBTBD4^P311PP^ treated with doxycycline compared to untreated samples. **C** Tumorsphere-forming capacity of D283Med cells in which KBTBD4^WT^, KBTBD4^R313PRR^ or KBTBD4^P311PP^ expression was induced with doxycycline (doxy+) compared to untreated controls (doxy−). Quantification of 3 independent experiments (bar chart) and representative pictures of doxycycline-induced cells (scale bar denotes 100 µm). **D** Aldehyde dehydrogenase (ALDH) activity, measured as percentage of ALDH positive cells, of D425Med parental cells or, D425Med cells expressing KBTBD4^WT^, KBTBD4^R313PRR^ or KBTBD4^P311PP^+/− LSD1i treatment (0.5 µM for 24 h). [N,N-diethylaminobenzaldehyde (DEAB), ALDH inhibitor]. *P* values (**P < 0.005 and ****P < 0.00005) were calculated by paired and two‐tailed *t*‐test.

To establish a causal link between tumorigenesis and KBTBD4^R313PRR^ or KBTBD4^P311PP^ expression, we measured cell proliferation of D283Med and D425Med after induction of KBTBD4^WT^, KBTBD4^R313PRR^ or KBTBD4^P311PP^. Surprisingly, we did not detect a significant difference in the proliferation of cells (Supplementary Fig. S5A), indicating that other features enable KBTBD4 mutants to promote tumorigenesis. In mice, genetic depletion of CoREST leads to the expansion of a progenitor stem cell population in the ventricular zone of the mouse brain with embryonic lethality [19]. Therefore, we hypothesised that CoREST degradation induced by KBTBD4 mutations could result in increased stemness of medulloblastoma cells, as described in other tumour types [20]. This hypothesis was supported by our RNA-seq data, wherein the expression of KBTBD4^R313PRR^ or KBTBD4^P311PP^ resulted in the enrichment of ES-like signature genes (Fig. 4A, B). Upon induction of KBTBD4^R313PRR^ or KBTBD4^P311PP^, cells expressed genes controlled by pluripotency factors such as SRY-Box Transcription Factor 2 (Sox2), Octamer-Binding Protein 4 (OCT4) and Homeobox Transcription Factor Nanog (Nanog) (Fig. 4A, B and Supplementary Table S2). In addition, Gene Set Enrichment Analysis (GSEA) showed that epithelial to mesenchymal transition (EMT), a fundamental pathway for vertebrate embryonic development and often linked to cell migration and metastatic potential of cancer cells [21], was significantly enriched in cell lines expressing either KBTBD4 mutant (Supplementary Fig. S5B). In adherent medulloblastoma cells, we also could detect an increased migration of cells expressing KBTBD4^P311PP^ (Supplementary Fig. S5C), supporting our GSEA analysis.

Most importantly, we assessed the effect of KBTBD4^WT^, KBTBD4^R313PRR^ and KBTBD4^P311PP^ expression on stemness using two well established assays for cancer stem cells, neurosphere formation ability and ALDH activity [22, 23]. Previous work showed that medulloblastoma initiating cells isolated based on ALDH activity had higher capacity for neurosphere formation, as well as higher expression of neural stem cell markers [24]. Additionally, these medulloblastoma initiating cells showed higher expression of pluripotency related genes and were able to be differentiated into multiple brain cell lineages [24]. Expression of KBTBD4^R313PRR^ and KBTBD4^P311PP^ induced increased neurosphere formation versus control D283Med cells, whereas expression of KBTBD4^WT^ did not (Fig. 4C). Note that the increased tumorsphere formation can’t be ascribed to increased cell proliferation as this remains unchanged (Supplementary Fig. S5A).

In a second assay to measure stemness, we observed that KBTBD4^R313PRR^ and KBTBD4^P311PP^ expression more than doubled the percentage of D283Med cells presenting with high ALDH activity compared to cells expressing KBTBD4^WT^ (Supplementary Fig. S5D). KBTBD4^R313PRR^ and KBTBD4^P311PP^ expression in D425Med cells also led to increased ALDH activity (Fig. 4D), showing that the stemness phenotype induced by KBTBD4^R313PRR^ and KBTBD4^P311PP^ overcomes *MYC* amplification present in the D425Med lines. More importantly, chemical inhibition of LSD1 (LSD1i) was found to mimic this phenotype and the combination of KBTBD4 mutant plus LSD1i did not show an additive effect (Fig. 4D and Supplementary Fig. S5C), showing that the increased stemness caused by KBTBD4^R313PRR^ and KBTBD4^P311PP^ is exerted via modulation of CoREST complex activity. Overall, our findings establish a potential mechanism of tumorigenesis through increased stemness in KBTBD4 mutant tumours.

### KBTBD4 transcriptional signatures define a specific cluster of medulloblastomas

**Fig. 5:**
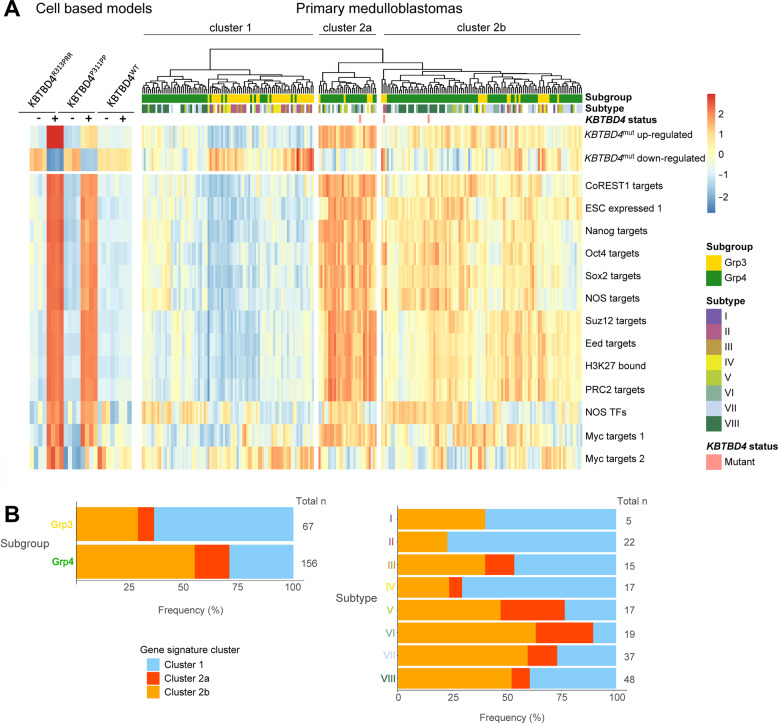
KBTBD4 transcriptional signatures define a specific cluster of medulloblastomas. **A** Heatmap showing single sample Gene Set Enrichment Analysis (ssGSEA) scores for D283Med cell-based models and human primary medulloblastomas (Group3; *n* = 67, Group4; *n* = 156). Custom genesets represented are dysregulated by doxocycline-induced expression of KBTBD4 mutants (R313PRR and P311PP) in D283Med cell lines. + indicates doxycycline treatment, - indicates untreated controls. **B** Stacked bar plots representing percentage frequency of gene signature clusters across different subgroups (Group3 and Group4) and subtypes (I-VIII) of primary medulloblastomas.

We next investigated the relevance of transcriptional programmes driven by KBTBD4^R313PRR^ and KBTBD4^P311PP^ in a large series of primary medulloblastomas. Using single sample Gene Set Enirchment Analysis (ssGSEA) of 223 primary MB_Group3/4_ tumours (Fig. 5A), interrogation of *KBTBD4* mutation-associated gene signatures identified in our models (including CoREST and ESC-related pathways) distinguished two clusters within these primary tumours. The first cluster displayed gene-set expression signatures similar to the KBTBD4^WT^ models, while signatures of the second tumour cluster were similar to those of the KBTBD4 mutant models. The second cluster further resolved into two sub-clusters (a,b), with sub-cluster ‘a’ displaying the strongest mutation-associated signatures. As hypothesised, all *KBTBD4* mutated primary tumours in the cohort (*n* = 3) resided in cluster 2 and demonstrated upregulation of *KBTBD4* mutation-associated genesets. Cluster 2 represents 60% (134/223) of all MB_Group3/4_ tumours examined, indicating a wider involvement of *KBTBD4* mutation-associated transcriptional programmes and CoREST pathways in this tumour group, with *KBTBD4*-mutated cases representing a subset (3/134) of these cases. Moreover, whilst cluster 2 tumours are seen in each molecular subgroup and subtype, they are particularly frequent in MB_Group4_ tumours (71% (110/156) of MB_Group4_), and their associated subtypes (V-VIII) (Fig. 5B).

## Discussion

**Fig. 6:**
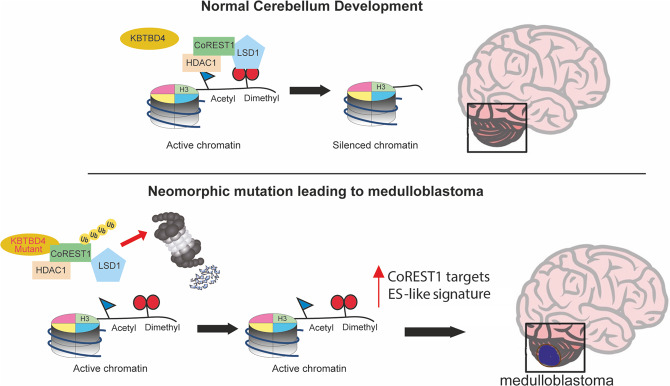
Neomorphic KBTBD4 mutations enable this E3 ubiquitin ligase to recruit, ubiquitylate and degrade CoREST favouring proliferation of neuronal progenitors and tumorigenesis. During normal development, CoREST complexes containing LSD1, HDAC1 or 2, and CoRESTs (also known as RCORs) silence chromatin by deacetylating and demethylating histone H3 at position Lys4 (K4). In medulloblastoma, mutations allow KBTBD4 to destabilise CoREST complexes thereby preventing chromatin repression leading to the increased transcription of CoREST targets and stemness genes. The activation of stemness genes is a potential driver of medulloblastoma tumorigenesis.

We have identified a novel pathogenetic mechanism in non-WNT/non-SHH medulloblastoma unveiling an actionable target. We find that oncogenic mutations in the ubiquitin ligase KBTBD4 in group 3/4 medulloblastomas lead to the recruitment and ubiquitylation of epigenetic regulatory complexes containing CoRESTs and LSD1. The lack of CoREST1/2 promotes the hyperproliferation of neuronal progenitors in the developing mouse brain [19]. Therefore, we surmise that the degradation of CoRESTs by neomorphic KBTBD4 mutants induces epigenetic remodelling that blocks neuronal precursor differentiation in favour of progenitor expansion (Fig. 6). This model uncovers a novel driver of epigenetic reprogramming in paediatric brain tumours, establishes a novel mode of tumorigenesis through gain-of-function mutations in ubiquitin ligases (neo-substrate recruitment) and identifies a new druggable target for tumour-specific therapies.

While mutations in ubiquitin ligases classically act in cancer by disrupting substrate recruitment, this study has defined the first prototypic example where E3 mutations promote the recruitment of neo-substrates. The change of substrate specificity in KBTBD4 raises the intriguing possibility that neomorphic mutations could be present in other E3 ligases, which would further expand the variety of degrons participating in cancer development that have been identified through deep learning algorithms [25]. In addition to the broad implications this observation has for the study of E3 ligases in cancer, it highlights a gain–of–function mutant E3 for the potential targeting of PROTAC drug modalities with tumour cell-specific activity.

Remarkably, the KBTBD4 mutations phenocopy the effects of the investigational drug UM171 [26], which is currently investigated in phase II clinical trials [27]. The authors proposed that UM171 may potentiate a weak interaction of the CoREST/LSD1 complex with the endogenous KBTBD4^WT^. However, our data obtained with 4 different models of medulloblastoma and HEK293T cells did not confirm binding of CoREST to KBTBD4^WT^, nor its ubiquitylation, suggesting a neomorphic effect in medulloblastoma cells resulting from KBTBD4 mutation.

CoREST acts as a core scaffold to recruit epigenetic remodellers and certain DNA-binding factors. We observed significant differences in the epigenetic marks H3K4 mono- and di-methylation consistent with altered LSD1 activity. We also observed a strong correlation between the KBTBD4-dependent degradation of CoREST and the transcriptional activation of CoREST target genes. While CoREST was first described as a partner of the RE1-silencing transcription factor REST, it has since been shown to assemble independently [28], leading to changes in its DNA site preferences and repressor activity. The precise function of CoREST therefore depends on the CoREST partner genes expressed and the subcomplexes formed. It has been shown previously that Growth Factor Independent Protein 1 (GFI1) and GFI1B are transactivated by enhancers in group 4 medulloblastoma and form a complex with LSD1 and CoREST that promotes tumour growth [29]. These observations are in stark contrast with the example of KBTBD4 where the loss of CoREST by ubiquitin-mediated degradation promotes stemness. However, the identified complex recruited by KBTBD4 did not include GFI1 or GFI1B likely explaining their different mechanisms. While GFI1 and GFI1B overexpressing tumours show high LSD1 activity that could be blocked using specific chemical inhibitors [30], our findings show that inhibition of LSD1 increases stemness (Fig. 4D and Supplementary Fig. S5D), raising caution over the use of LSD1 inhibitors in the treatment of specific subtypes of medulloblastoma. Our data also indicate that KBTBD4 mutation leads cells into an intermediate state of differentiation, keeping ES-like and EMT gene signatures (Fig. 4A, B and Supplementary Fig. S5A) while expressing differentiated features of several synaptic pathways (Fig. 3D, E). This is in line with evidence showing that embryonic stem cells lacking LSD1 fail to fully differentiate [31].

Data from our human primary tumour cohorts support our cell-based findings. Human medulloblastomas with CoREST signatures showed an increase in expression of well-established stemness-associated transcription factor gene-sets (e.g. Nanog, Oct4, Sox2) (Fig. 5A). Moreover, these expression signatures define a cluster of tumours which include, but extend beyond, KBTBD4 mutant cases to characterise a wider disease group. Together, these initial findings suggest a wider involvement, and open further lines of investigation, of the role of CoREST in non-WNT/non-SHH medulloblastoma.

Neomorphic mutations are increasingly implicated as a molecular driver of tumorigenesis, including brain tumours. Perhaps the most established examples are the mutations found in in Isocitrate Dehydrogenase 1 (IDH1) and IDH2 that cause a major subset of gliomas. These mutations alter the enzyme activity to convert alpha-ketoglutarate into 2-hydroxyglutarate, which acts as an epigenetic remodeller to inhibit glioma stem cell differentiation [32]. The discovery of neomorphic mutations in KBTBD4 extends the spectrum of neomorphic mutations in cancer, highlighting KBTBD4 and the CoREST complex for further investigations of the origins and therapeutic opportunities in group 3/4 medulloblastomas.

## Materials and Methods

### Cell Lines

HEK293T cells were cultured in high glucose Dulbecco’s Modified Eagle’s Medium (GIBCO #41965039) with 5% Penicillin Streptomycin (ThermoFisher Scientific # 15070063) and 10% Fetal Bovine Serum (Sigma-Aldrich #F9665) inside a 5% CO_2_ incubator at 37 °C. D283Med (ATCC® HTB-185™) and DAOY (ATCC® HTB-186™) cell lines were purchased from ATCC. D425Med, D458Med and HDMB-03 were gifts from Professor Beth Coyle, the University of Nottingham. All medulloblastoma cells were cultured in Minimum Essential Medium (MEM) (Thermo Fisher Scientific #11095080) supplemented with 10% Fetal Bovine Serum (Sigma-Aldrich #F9665) inside a 5% CO_2_ incubator at 37 °C. D283Med and D425Med cells infected with pCW57.1 were cultured in MEM (Thermo Fisher Scientific #11095080) supplemented with 10% Tetracycline-Free Fetal Bovine Serum (PAN Biotech # Fetal Bovine Serum).

### Plasmids and gene transfer

For studies conducted in HEK293T cells, full length human KBTBD4 (Uniprot Q9NVX7 isoform 1) was cloned into pcDNA3.1(+) with the 3xFLAG epitope tagged to its N-terminus using standard restriction enzyme cloning. The mutant KBTBD4 constructs (R313PRR and P311PP) were created in the same vector by site directed mutagenesis. DNA sequences were verified by Source Bioscience Ltd. CoREST1 (pCMV5 HA RCOR1; NM_015156.4) construct was purchased from the MRC Protein Phosphorylation and Ubiquitylation Unit (https://mrcppureagents.dundee.ac.uk). Myc-His-Ubiquitin plasmid was used as previously described [1, 2]. For studies conducted in D283Med and D425Med cell lines, Flag-KBTBD4 wild type and mutants were first cloned into pENTR3C (ThermoFisher Scientific #A10464) and then transferred through GATEWAY (Thermo Fisher Scientific #11791100) into pCW57.1 (Addgene #41393). For studies conducted in the DAOY cell line, Flag-KBTBD4 wild type and mutants were cloned into MIGR1 (Addgene #27490). pCW57.1 was a gift from David Root (Addgene plasmid # 41393; http://n2t.net/addgene:41393; RRID:Addgene_41393).

pCW57.1 and MIGR1 virus were produced in HEK293 cells using packaging plasmids psPAX2 and pMD2G and Transfection Reagent TransIT®-Lenti (MIR 6610). Media was replaced after 16 h and virus production was allowed for 48 h. Media containing virus was collected, filtered, supplemented with 10 ng/mL polybrene and medulloblastoma cells lines were infected. After 16 h, media was replaced by complete media and cells were allowed to recover for 48 h before antibiotic selection.

### Sample preparation for mass spectrometry proteomics

Flag-KBTBD4 wild type or mutants along with an empty vector control were transfected into HEK293T cells at 60% confluency with PEI-MAX 40kDA (11 µg DNA with 66 µg PEI per 15 cm dish). 40 h post transfection, cells were treated with 100 nM MLN4924 (Calbiochem, 951950–33–7) for 4 h before harvesting. Cells were then washed with ice-cold PBS and lysed in lysis buffer (50 mM Tris pH 7.5, 150 mM NaCl, 10% glycerol, 0.1% NP-40, 1 mM EDTA and 5 mM MgCl_2_) containing protease inhibitors (Sigma-Aldrich), beta-glycerolphosphate, DTT, PMSF and okadaic acid. 25 mg protein extract per sample was precleared with protein A agarose (Invitrogen), and then immunoprecipitated by ANTI-FLAG M2 Affinity Gel (Sigma-Aldrich). Flag-KBTBD4 and its interactors were eluted with 3xFLAG peptide (Sigma-Aldrich). The FLAG eluates were first incubated with 5 mM DTT for 30 min for reduction and then with 20 mM iodoacetamide for 30 min for alkylation. Then the proteins in the eluates were precipitated by methanol/chloroform to remove organic impurities. Precipitated proteins were reconstituted in 20 mM HEPES pH 8, 8 M urea, and then diluted to 20 mM HEPES pH 8, 1 M urea before adding immobilised trypsin resin (Pierce 20230) for 16 h at 37 °C. Trypsin digestion was terminated by adding 1% TFA and trypsin resin removed by centrifugation. Tryptic-digested peptides were desalted using the SOLA HRP SPE cartridges (Thermo Fisher Scientific) and dried using a vacuum spinner (Thermo Fisher Scientific).

### LC‐MS/MS and data analysis

Dried tryptic-digested peptides were re-constituted in 15 µL of LC-MS grade water containing 1% acetonitrile and 0.1% TFA. Samples were analysed by liquid chromatography-tandem mass spectrometry (LC-MS/MS) using a Dionex Ultimate 3000 UPLC coupled to a Q-Exactive mass spectrometer (Thermo Fisher Scientific). The peptides were loaded onto a trap column (PepMapC18; 300 µm × 5 mm, 5 µm particle size, Thermo Fischer Scientific) for 1 min at 20 μL/min flowrate. The loaded peptides were separated on a 50 cm-long chromatographic EasySpray column (ES803, Thermo Fischer Scientific) with a gradient of 2 to 35% acetonitrile in 0.1% formic acid and 5% DMSO at 250 nL/min flow rate for 60 min. The Q-Exactive was operated in a data-dependent acquisition (DDA) mode to automatically switch between full MS-scan and MS/MS acquisition. Survey-full MS scans were acquired in the Orbitrap mass analyser over an m/z window of 380–1500 and at a resolution of 70k (AGC target at 3e6 ions). Prior to MSMS acquisition, the top fifteen most intense precursor ions (charge state ≥ 2) were sequentially isolated in the Quad (m/z 1.6 window) and fragmented on the HCD cell (normalised collision energy of 28). MS/MS data were obtained in the orbitrap at a resolution of 17.5 K with a maximum acquisition time of 128 ms, an AGC target of 1e5 and a dynamic exclusion of 27 s.

The raw data were searched against the Human UniProt-SwissProt database (Jan 2018; containing 20,259 human sequences) using the Mascot data search engine. The search was carried out by enabling the Decoy function, whilst selecting trypsin as enzyme (allowing 1 missed cleavage), peptide charge of +2, +3, +4 ions, peptide tolerance of 10 ppm and MS/MS of 0.05 Da; #13 C at 1; Carboamidomethyl (C) as fixed modification; and Deamidated (NQ), Oxidation (M), as a variable modification. MASCOT outputs were filtered using an ion score cut off of 20 and a false discovery rate (FDR) of 1%. The mass spectrometry raw data included in this paper had been deposited to the Proteome eXchange Consortium via the PRIDE partner repository with the dataset identifier PXD031683 [3].

### Immunoprecipitation and immunoblotting

HEK293T cells were transfected as indicated at 60% confluency (5 µg DNA per plate using PEI-MAX 40kDA at ratio 6:1). After 20 h, cells were treated with 0.1 µM MLN4924 (Calbiochem, 951950–33–7) for 4–5 h. Cells were washed twice with ice-cold PBS, harvested into lysis buffer (50 mM Tris pH 7.5, 150 mM NaCl, 10% glycerol, 0.1% NP-40, 1 mM EDTA and 5 mM MgCl_2_) containing protease inhibitors (Sigma-Aldrich), beta-glycerolphosphate, DTT, PMSF and okadaic acid, incubated on ice for 30 min and centrifuged for 30 minutes at 20 000 g at 4 °C. The supernatant was incubated with ANTI-FLAG M2 Affinity Gel (Sigma-Aldrich) or HA affinity gel (E6779–1ML, Sigma-Aldrich) at 4 °C for 1 h before being washed 4 times with lysis buffer. Co-immunoprecipitated samples were eluted using 2× LDS buffer (Life Technologies) supplemented with β-mercaptoethanol and boiled for 5 min at 95 °C.

D283med cells harbouring pCW57.1 KBTBD4 variants were induced with 1 µg/mL doxycycline for 40 hours before 6 h 0.1 µM MLN4924 (Calbiochem, 951950–33–7) treatment as indicated in the figure. For each dish, adherent cells were scraped off and combined with cells in suspension collected by centrifugation. Cells were washed and lysed the same way as described above. The supernatant was incubated with ANTI-FLAG M2 Affinity Gel (Sigma-Aldrich) at 4 °C for 3 h. After 4 times of washes with lysis buffer, co-immunoprecipitated samples were eluted in LDS buffer (Life Technologies) supplemented with β-mercaptoethanol and boiled for 5 min at 95 °C. Whole-cell lysates were obtained by lysing cells in 2xSB (625 mM Bis-Tris pH 6.8, 20% (w/v) glycerol, 4% (w/v) SDS), boiled, sonicated and protein concentration was evaluated using BCA protein kit (Thermo Fisher Scientific) in most cases. Cell lysate or immunoprecipitate was resolved in 10% Bis-Tris gels, transferred to nitrocellulose membranes (Millipore) and immunoblotted with corresponding antibodies. The same membranes were probed with Anti-GAPDH (Thermo Fisher Scientific MA5–15738) or Anti-alpha Tubulin (TU-02) antibody (Santa Cruz Biotechnology, # sc-8035) for loading control.

These techniques have been described previously and are detailed above [33–35]. Uncropped Western blots are provided in Supplementary Material.

### Recombinant protein production of GST-UBA^Ubq^

An expression plasmid encoding the UBA domain of Ubiquilin-1 (Q9UMX0, isoform 1, 536–589 a.a.) fused with N-terminal GST and C-terminal hexahistidine tags in pGEX6P1 was obtained as a gift from Prof Mads Gyrd-Hansen’s lab in the University of Oxford [4]. The plasmid was transformed into E.coli strain BL21(DE3)-R3-pRARE2. Cells were cultured in TB medium at 37 °C until OD_600_ reached 2. Recombinant UBA^Ubq^ expression was then induced by addition of 0.4 mM isopropyl β-D-1thiogalactopyranoside, followed by 18 h continuous shaking at 18 °C. Cells were harvested by centrifugation and lysed by sonication in binding buffer (50 mM HEPES pH 7.5, 500 mM NaCl, 5% glycerol, 5 mM imidazole) supplemented with 0.5 mM TCEP. UBA^Ubq^ was captured on nickel sepharose resin, washed with binding buffer and eluted by a stepwise gradient of 30–250 mM imidazole. The protein was further cleaned up by size exclusion chromatography using a HiLoad 16/60 S200 Superdex column buffered in 50 mM HEPES pH 7.5, 300 mM NaCl, 0.5 mM TCEP. Protein masses were confirmed by intact LC-MS mass spectrometry.

### Ubiquitylation assay

HEK293T cells at 60% confluency were transfected with 1 µg HA-CoREST1, 1 µg Myc-Ubiquitin and 1 µg Flag KBTBD4 wild type or mutants along with empty vector control as indicated per 10 cm dish. 30 hours after transfection, cells were washed with ice-cold PBS once. Cells were then thoroughly lysed and boiled in 500 μL denaturing lysis buffer (2% SDS, 150 mM NaCl, 10 mM Tris-HCl pH 7.4, supplemented with protease inhibitors). After cooling down to room temperature, cell lysates were then subjected to sonication until losing viscosity. Lysates were then diluted 10 times with dilution buffer (10 mM Tris-HCl pH 7.4, 150 mM NaCl, 2 mM EDTA, 1% Triton x-100) and incubated at 4 °C for 30 min. The diluted lysates were centrifuged at 14,000 rpm for 10 min to remove insoluble debris. Supernatant was used for immunoprecipitation with HA affinity gel (E6779–1ML, Sigma-Aldrich, 10 μL beads per sample) overnight at 4 °C. HA affinity gel was collected via centrifugation at 200 × *g* for 90 s and washed with 1 mL wash buffer (10 mM Tris-HCl pH 7.4, 1 M NaCl, 1 mM EDTA, 1% NP-40) 4 times before being mixed with 50 μL LDS buffer (Life Technologies) and boiled. Results were analysed by immunoblotting (Anti-Flag antibody, Sigma-Aldrich #F1804; Anti-HA antibody, Roche #12013819001; Anti-Myc antibody, Cell Signaling Technology #9B11).

D283Med cells harbouring Flag-KBTBD4 variants were induced with 1 μg/mL doxycycline for 10 hours where indicated followed by 6 h of 5 μM MG132 treatment along with the parental D283Med cells. Cells were lysed in buffer containing 20 mM sodium phosphate buffer pH 7.4, 1% NP40, 2 mM EDTA supplemented with 5 mM N-ethylmaleimide, 50 μM PR619 and protease inhibitors. 150 µg/mL UBA^Ubq^ was incubated with glutathione sepharose 4B resin (GE Healthcare) for at least 1 hour and then the unbound protein was washed away.

Endogenous ubiquitylated proteins were pulled down by glutathione sepharose resin pre-bound with UBA^Ubq^ and eluted with LDS buffer. Results were analysed by immunoblotting (Anti-Flag antibody, Sigma-Aldrich #F1804; Anti-GAPDH antibody, Thermo Fisher Scientific MA5–15738; Anti-CoREST1, Cell Signaling Technology #14567).

### RNA-sequencing

1 × 10^6^ D283Med cells harbouring Flag‐KBTBD4^WT^, Flag-KBTBD4^R313PRR^ or Flag-KBTBD4^P311PP^+/− doxycycline (1 µg/mL) were cultured for 4 days before harvesting. After washing once in phosphate-buffered saline, cell pellets were resuspended in TRIzol Reagent (Thermo Fisher Scientific cat # 15596026), snap frozen and preserved at −80 °C. RNA extraction, library preparation and sequencing were outsourced to NOVOGENE.

### Small interfering RNAs transfection

Silencer® Select Small interfering RNAs (siRNA) (Thermo Fisher Scientific) targeting CoREST1 (siRNA 1 ID s23229 and siRNA 2 ID s23230) were transfected in D283Med and D425Med cells using HiPerFect Transfection Reagent (Qiagen). Cells were harvested 72 h after transfection and used for WB analysis of knockdown efficiency and Total RNA extraction for gene expression analysis.

### Total RNA extraction and cDNA synthesis

Total RNA from medulloblastoma cell lines was isolated using RNeasy Plus Micro Kit (Qiagen, #74034) following the manufacturer’s protocol. RNA with an absorbance read 260/280 ratio between 1.8 and 2.0, assessed on a Nanodrop2000 spectrophotometer (Thermo Fisher Scientific), was used for reverse transcription. For every sample, 0.5 µg of total RNA was used for cDNA synthesis using SuperScript IV VILO Master Mix (Thermo Fisher Scientific, #11756050) following the manufacturer’s protocol.

### qPCR

For real time gene expression quantification, PowerTrack™ SYBR Green Master Mix (Thermo Fisher Scientific, # A46113) was used. Specific pair of primers were designed for each gene transcript using Primer designing tool - NCBI (http://bioinfo.ut.ee/primer3–0.4.0/https://www.ncbi.nlm.nih.gov/tools/primer-blast/), and pair of primers analysis to predict secondary structures formation was done by using Beacon Designer tool (http://www.premierbiosoft.com/qOligo/Oligo.jsp?PID=1). The list of primers used in this study is reported below. All reactions were run in QuantStudio™ 5 Real-Time PCR System, 384-well (Thermo Fisher Scientific, #A28140). Each sample was tested in triplicate. Data analysis was performed with Design & Analysis software version 2.5.1 (Thermo Fisher Scientific). Relative changes in expression (i.e doxycycline treated relative to untreated controls) were evaluated by using the 2ΔCt formula.

**Table:** 

Assay	Gene symbol	Accession Number	Forward primer 5’-3’	Reverse primer 5’-3’
qPCR	ALPL	NM_001127501	AACATCAGGGACATTGACGTG	GTATCTCGGTTTGAAGCTCTTCC
	AMIGO2	NM_181847	CCTGGGAACCTTTTCAGACTG	GCAAACGATACTGGAATCCACT
	CLU	NM_001831	CTACTTCTGGATGAATGGTGACC	CGGGTGAAGAACCTGTCCT
	CLVS2	NM_001010852	TACACACTGGTGGATATTTTGCG	TTTAGAGGCTTGCTTGAAAGTGA
	COL6A2	NM_058174	GAACGGGACCGATGGACAG	CCCTTGGCCCCGATTTCTC
	HEPH	NM_001130860	TGCGATATGAAGCCTTTCAAGAT	GGAGGCACGGTTGTAGAAGA
	RAB3B	NM_002867	CAACAGCCTATTACCGTGGGG	TAGCCCAGTCTTGGACAGCA
	SCN1A	NM_001202435	ATGTGGAAATAGCTCTGATGCAG	AGCCCAACTGAAGGTATCAAAG
	SCN9A	NM_002977	ATTCGTGGCTCCTTGTTTTCTG	CTACTGGCTTGGCTGATGTTAC
	SFRP1	NM_003012.5	CTCCATAGCCACGCTCCAAA	TCTCACTTTCCGCCCAATCC
	SPARC	NM_003118	CCCATTGGCGAGTTTGAGAAG	CAAGGCCCGATGTAGTCCA
	STC1	NM_003155	CACGAGCTGACTTCAACAGGA	GGATGTGCGTTTGATGTGGG
	TGM2	NM_004613	CAAGGCCCGTTTTCCACTAAG	GAGGCGATACAGGCCGATG
	TJP3	NM_014428	GCTTTGGCATTGCGATCTCTG	GATGTGGTCGCCTGTCTGTAG
ChIP-qPCR	CLU	NM_001831	GAGCCAGCACAGCTATTCGT	CCAAAGAATGCCGCGGAAAG
	CLVS2	NM_001010852	CTTGCACACAAAGGGCGAAG	GGTAGGACGGCAATTTGGGT
	COL6A2	NM_058174	CTGAGCAAGCCGGACACA	GACTCGCCCCTTGGTAGC
	HEPH	NM_001130860	AAGGGGAAGAGGTGGTGAGA	GGGGGTGGGTTGTACTTCTG
	SCN9A	NM_002977	TCGCTCCTACCAGCTCTGAA	GGGGAAAGAAACGTGGGGAA

### Chromatin immunoprecipitation

D283Med and D425Med cells harbouring Flag‐KBTBD4^WT^ or Flag-KBTBD4^R313PRR^ −/+doxycycline (1 µg/mL) were cultured for 3 days before the assay. Approximately 4 × 10^6^ cells for each immunoprecipitation was processed using SimpleChIP® Plus Enzymatic Chromatin IP Kit (Cell Signalling Technology, #9005) following the manufacturer’s recommended protocol. We performed the optimisation of chromatin digestion recommended and 0.5 µL of Micrococcal Nuclease per IP prep was used (20 min at 37^o^C). Normal Rabbit IgG (#2729), Histone H3 (#4620) and LSD1 (#2184) antibodies from Cell Signalling Technology were used at the manufacturer’s recommended concentration. qPCR was performed and analysed as described above using 2 µL of purified ChIP DNA. Data were plotted as percentage of input.

### Tumorsphere assays

Tumorsphere formation assays were performed as described previously [22]. Tumoursphere formation assays were performed in Ultra-Low Attachment 6-wells plates (Costar^®^ #CLS3471) or cell-repellent surface 6-wells plates (Cellstar^®^ #657970). D283Med cells were plated at a density of 5000 cells per well in triplicate, with or without doxycycline (1 µg/mL) in 1% methylcellulose containing media (NeuroCult^TM^ #05751, supplemented with MethoCult^TM^ #04100, STEMCELL Technologies). Tumourspheres were imaged and quantified 7 days after plating using a Celigo Image Cytometer (Nexcelom Bioscience LLC). Spheres with a diameter equal or higher than 40 μm were deemed tumourspheres. Experiments were repeated at least three times.

### ALDH activity

ALDH activity was measured in intact D283Med cells using the ALDEFLUOR™ (STEMCELL Technologies, #01700) following the manufacturer’s protocol. One million D283Med cells were resuspended in 1 mL of Aldefluor assay buffer and mixed with 5 µL of BODIPY-aminoacetaldehyde (BAAA) ALDH substrate. Immediately after the addition of BAAA, half of the cells were transferred into a tube containing 10 µL of the ALDH inhibitor N,N-diethylaminobenzaldehyde (DEAB). After a 30-min incubation period, cells were pelleted to discard supernatant and then resuspended in 250 µL of Aldefluor assay buffer. Cells were analysed using a CytoFLEX LX Flow Cytometer, and results were generated using FlowJo software. Experiments were repeated at least three times.

### Flow cytometry

For the detection of H3K4 mono and di-methylation in D283Med, cells were dissociated to single cells, pelleted and washed once in phosphate-buffered saline. Fixation and permeabilization were performed using the True Nuclear fixation kit (BioLegend, Cat #424401) following the manufacturer recommendations. Cells were then stained with rabbit anti-H3K4me1 (Cell Signaling Technology, Catalog #5326), rabbit anti-H3K4me2 (Cell Signaling Technology, Catalog #9725), normal rabbit IgG (Isotype control, sc-2027) and Alexa Fluor 594 (Thermo Fisher Scientific # A-21207). Flow cytometry analysis was performed using BD LSR Fortessa X20 (Becton Dickinson, San Jose, CA, USA) using the 488 nm laser (60 mW) for FSC (380 V) and SSC (212 V), and 561 nm laser (50 mW) using a 610/20 nm bandpass filter and a PMT voltage of 390 for AF594 excitation/detection. Results were generated using FlowJo software. Negative and Positive gates were determined using 5 controls: 1. Compensation beads (AbC™ Total Antibody Compensation Bead Kit, Thermo Fisher Scientific #A10513) composed by: a) polystyrene microspheres that have primary/secondary fluorescent antibody conjugate capture capacity (positive compensation beads) to define a clear positive signal and; b) inert beads (negative compensation beads) to provide a clear negative signal; 2. Unstained cells used to detect “auto-fluorescence” of the cells of interest and define negative gating; 3. Cells stained only with secondary antibody used to detect background staining and define negative gating; 4. Cells treated with LSD1i following incubation either with: a) isotype primary antibody and secondary antibody (used to define negative gating) or; b) H3K4me2 primary antibody and secondary antibody (used to define positive gating). Additionally, species and concentration matching isotype control was used for all primary antibodies and sample type.

### Structural modelling

Homology models of the Kelch domain of KBTBD4 and its mutants were prepared using the ICM software package (Molsoft) [5] based on crystal structures of previously solved Kelch domains. The initial model was refined by energy minimisation and side chain optimisation in ICM-Pro (Molsoft).

### Migration assay

A scratch wound assay was used to measure the migration capacity of DAOY cells overexpressing KBTBD4 wild type or mutants. Cells were seeded at 3 × 10^5^ per well into a 12-wells plate in starvation medium with 1% FBS to form a confluent cell monolayer. Then, using a sterile 20 μL plastic pipette tip, two linear scratches (cross shape) were generated in each well. Media were carefully aspirated to remove floating cells generated by the scratch and replaced by a complete growth media (MEM supplemented with 10% FBS). Images were acquired every 2 h for a period of 24 h using a Zeiss LSM 780 confocal microscope equipped with live cell imaging incubator system.

### Quantification and statistical analysis

RNA sequencing reads were aligned to the reference GRCh38 using HISAT2 v2.0.5. StringTie v1.3.1 was used to assemble transcripts and estimate transcript abundance in each sample. Gene FPKMs (fragments per kilo-base of exon per million fragments mapped) were computed by summing the FPKMs of transcripts in each gene group. Differential expression analysis was calculated using Ballgown at transcript level [36]. Transcripts with *P*-adjust < 0.05 were described as differentially expressed between any two groups. Gene level counts were computed using StringTie and DESeq2 v1.32.0R package was used for differential expression analysis [37]. KEGG pathway analysis were performed using the clusterProfiler v 3.13.0 [8] KEGG with Benjamini Hochberg (BH) corrected *P* value >0.05 were considered as significantly enriched. EnrichR was used to perform transcription factor analysis and identify CoREST and SOX2/NANOG/OCT4 lists of genes differentially expressed (upregulated) in KBTBD4 mutants. *P* values <0.05 were considered significant [38–40].

Analysis of human medulloblastoma tumours was carried out using RNA sequencing datasets [41]. For clustering and visualisation, read counts were normalised and a variance stabilising transformation applied using ‘vst’ function (DESeq2, R/Bioconductor). Single sample Gene Set Enrichment Analysis (ssGSEA) was performed using the implementation within GSVA (R/Bioconductor) and heatmaps produced using ‘pheatmap’ (R/Bioconductor). Mutations within *KBTBD4* hotspot Kelch motif region (codons 308–313) were identified by direct PCR, Sanger sequencing and visual inspection of electropherograms.

### Materials availability

Reagents generated in this study will be made available on request. A completed Materials Transfer Agreement may be requested if there is potential for commercial application.

### Table of reagents

**Table:** 

Reagent or resource	Source	Identifier
Antibodies		
Anti-FLAG antibody	Sigma-Aldrich	Cat#F1804; RRID:AB_262044
Anti-FLAG M2 Affinity Gel	Sigma-Aldrich	Cat#A2220; RRID:AB_10063035
Anti-HA-Peroxidase antibody	Roche	Cat#12013819001, RRID:AB_390917
EZview^TM^ Red Anti-HA Affinity Gel	Sigma-Aldrich	Cat#E6779, RRID:AB_10109562
Anti-Myc antibody	Cell Signaling Technology	Cat#2276, RRID:AB_331783
Anti-LSD1 antibody	Cell Signaling Technology	Cat#2184, RRID:AB_2070132
CoREST1 (D6I2U) Rabbit mAb	Cell Signaling Technology	Cat#14567, RRID:AB_2798514
alpha Tubulin (TU-02) antibody	Santa Cruz Biotechnology	Cat# sc-8035, RRID:AB_628408
Mono-Methyl-Histone H3 (Lys4) (D1A9) Rabbit mAb	Cell Signaling Technology	Cat# 5326, RRID:AB_10695148
Di-Methyl-Histone H3 (Lys4) (C64G9) Rabbit mAb	Cell Signaling Technology	Cat# 9725, RRID:AB_10205451
Histone H3 (D1H2) XP® Rabbit mAb	Cell Signaling Technology	Cat# 4499, RRID:AB_10544537
Normal Rabbit IgG	Cell Signaling Technology	Cat#2729, RRID:AB_1031062
Normal rabbit IgG (Isotype control)	Santa Cruz Biotechnology	Cat# sc-2027, RRID:AB_737197
Donkey anti-Rabbit IgG (H + L) Highly Cross-Adsorbed Secondary Antibody, Alexa Fluor 594	Thermo Fisher Scientific	Cat# A-21207, RRID:AB_141637
Anti-GAPDH antibody	Thermo Fisher Scientific	Cat#MA5–15738, RRID:AB_10977387
Anti-Cul3 antibody	Bethyl	Cat#A301–109A, RRID:AB_873023
Bacterial and virus strains		
Subcloning Efficiency™ DH5α Competent Cells	Thermo Fisher Scientific	Cat# 18265017
One Shot™ ccdB Survival™ 2 T1R Competent Cells	Thermo Fisher Scientific	Cat# A10460
One Shot™ Stbl3™ Chemically Competent E. coli	Thermo Fisher Scientific	Cat# C737303
Chemicals, peptides, and recombinant proteins		
3xFLAG peptide	Sigma-Aldrich	Cat#F4799
Immobilised trypsin	Pierce	Cat#20230
Neddylation inhibitor MLN4924	Calbiochem	Cat#951950–33–7
Proteasome inhibitor MG132	ApexBio Technology	Cat#A2585
Doxycycline	Sigma-Aldrich	Cat#D3447
Puromycin	Life technologies	Cat#A1113803
PR-619	Selleck Chem	Cat#S7130
NEM	Sigma-Aldrich	Cat#E3876
TUBE Agarose resin	Lifesensors	Cat# UM-0402
GSK-LSD1 2HCl (LSD1 inhibitor)	Selleckchem	Cat#S7574
PEI Max 40KDA	Polysciences	Cat# 24765
HiPerFect Transfection Reagent	Qiagen	Cat# 301704
OptiMEM	GIBCO	Cat#31985070
NeuroCult^TM^ Neural Cell Culture Media	STEMCELL Technology	Cat#05751
MethoCult^TM^	STEMCELL Technology	Cat#04100
MEM (Minimum Essential Medium)	Thermo Fisher Scientific	Cat#11095080
Dulbecco’s Modified Eagle’s Medium	GIBCO	Cat#41965039
Fetal Bovine Serum	Sigma-Aldrich	Cat#F9665
Fetal bovine serum, EU approved, Tetracycline Free	PAN Biotech	Cat# P30–3602
Critical commercial assays		
ALDEFLUOR™	STEMCELL Technologies	Cat#01700
True Nuclear fixation kit	BioLegend	Cat # 424401
AbC™ Total Antibody Compensation Bead Kit	Thermo Fisher Scientific	Cat#A10513
Q5 Site-Directed Mutagenesis Kit	New England Biolabs	Cat# E0554S
TransIT®-Lenti	MirusBio	Cat# MIR 6610
Gateway™ LR Clonase™ II Enzyme mix	Thermo Fisher Scientific	Cat#11791100
SimpleChIP® Plus Enzymatic Chromatin IP Kit	Cell Signaling Technology	Cat#9005
PowerTrack™ SYBR Green Master Mix	Thermo Fisher Scientific	Cat# A46113
RNeasy Plus Micro Kit	Qiagen	Cat#74034
SuperScript IV VILO Master Mix	Thermo Fisher Scientific	Cat#11756050
Deposited data		
Structures used for KBTBD4 Kelch domain homology modelling	https://rcsb.org	PDB: 1U6D, 4ASC, 4CH9, 6GY5
Experimental models: cell lines		
HEK293T	ATCC	Cat#CRL-3216; RRID:CVCL_0063
D283Med	ATCC	Cat# HTB-185, RRID:CVCL_1155
D425Med	Millipore	Cat# SCC290, RRID:CVCL_1275
D458Med		
HD-MB03	DSMZ	Cat# ACC-740, RRID:CVCL_S506
DAOY	ATCC	Cat# HTB-186, RRID:CVCL_1167
Oligonucleotides		
KBTBD4 pcDNA3.1(+) fwd: CCGGCCGGATCCATGGAATCACCAGAGGAGCCTGG	Eurofins Genomics	N/A
KBTBD4 pcDNA3.1(+) rev: GCATACGTCGACTTAGGCCAACACAAACTGCAAATTG	Eurofins Genomics	N/A
KBTBD4 R313PRR site direct mutagenesis fwd: TTGTATGTGGTGGGAGGGTCCATCCCACGGCCACGGCGCATGTGGAAGT	Eurofins Genomics	N/A
KBTBD4 R313PRR site direct mutagenesis rev: ACTTCCACATGCGCCGTGGCCGTGGGATGGACCCTCCCACCACATACAA	Eurofins Genomics	N/A
KBTBD4 P311PP site direct mutagenesis fwd: TTGTATGTGGTGGGAGGGTCCATCCCACCACGGCGCATGTGGAAGT	Eurofins Genomics	N/A
KBTBD4 P311PP site direct mutagenesis rev: ACTTCCACATGCGCCGTGGTGGGATGGACCCTCCCACCACATACAA	Eurofins Genomics	N/A
KBTBD4 MIGR1 fwd: CCGGCCGGATCCATGGACTACAAAGACGATGACGACAAGGAATCACCAGAGGAGCCTGG	Thermo Fisher Scientific	N/A
KBTBD4 MIGR1 rev: GCATACGTCGACTTAGGCCAACACAAACTGCAAATTG	Thermo Fisher Scientific	N/A
KBTBD4 pCW57.1 fwd: GGGGGGTCGACCCACCATGGACTACAAAGACGATGACGACAAGGAAT	This paper	N/A
KBTBD4 pCW57.1 Rev: GGGGGCTCGAGTTAGGCCAACACAAACTGCAAATTGGTAAGC	This paper	N/A
Silencer® Select Small interfering RNAs (siRNA) targeting CoREST1	Thermo Fisher Scientific	siRNA 1 ID s23229 siRNA 2 ID s23230
Recombinant DNA		
pcDNA3.1 (+) FLAG-KBTBD4 full-length wild type	This paper	N/A
pcDNA3.1 (+) FLAG-KBTBD4 full-length R313PRR	This paper	N/A
pcDNA3.1 (+) FLAG-KBTBD4 full-length P311PP	This paper	N/A
pCMV5 HA RCOR1 (custom)	MRC Protein Phosphorylation and Ubiquitylation Unit	N/A
MIGR1 FLAG-KBTBD4 full-length wild type	This paper	N/A
MIGR1 FLAG-KBTBD4 full-length R313PRR	This paper	N/A
MIGR1 FLAG-KBTBD4 full-length P311PP	This paper	N/A
pCW57.1 FLAG-KBTBD4 full-length wild type	This paper	N/A
pCW57.1 FLAG-KBTBD4 full-length R313PRR	This paper	N/A
pCW57.1 FLAG-KBTBD4 full-length P311PP	This paper	N/A
pENTR3C FLAG-KBTBD4 full-length wild type	This paper	N/A
pENTR3C FLAG-KBTBD4 full-length R313PRR	This paper	N/A
pENTR3C FLAG-KBTBD4 full-length P311PP	This paper	N/A
Software and algorithms		
Molsoft ICM-Pro	Molsoft LLC.	http://www.molsoft.com/icm_pro.htmlICM Pro
Mascot search engine	Matrix Science	https://www.matrixscience.com/search_form_select.html

## Supplementary information

Supplementary Figures and legends

Original Western blot images

Checklist for CDD

Supplementary Table S1

Supplementary Table S2

## Data Availability

LC/MS-MS raw data are available through https://www.ebi.ac.uk/pride with the following accession number: PXD031683. RNA-seq data have been deposited in GEO with the following accession number: GSE197240.
