# Supplementary material for: Disease-associated KBTBD4 mutations in medulloblastoma elicit neomorphic ubiquitylation activity to promote CoREST degradation
Source: Cell Death Differ. 2022 Apr 4;29(10):1955–69. doi: 10.1038/s41418-022-00983-4 (PMC9525703; doi:10.1038/s41418-022-00983-4)
Supplement: Supplementary file 1 — Supplementary Figures and legends [file 41418_2022_983_MOESM1_ESM.pdf]

Disease-associated KBTBD4 mutations in medulloblastoma elicit neomorphic ubiquitylation activity to promote CoREST degradation

Zhuoyao Chen, Rafael M Ioris, Stacey Richardson, Ava N Van Ess, Iolanda Vendrell, Benedikt M Kessler, Francesca M Buffa, Luca Busino, Steven C Clifford, Alex N Bullock and Vincenzo D'Angiolella

Supplementary Figures S1-S5 and legends

KBTBD4

```
Homo sapiens (Human)        304-YVVGGSIPRRMWKCNNA
Mus musculus (Mouse)        313-YVVGGSIPRRMWKCNNA
Gallus gallus (Chicken)     313-YVVGGSIPRRMWKCNNA
Xenopus tropicalis (Frog)   386-YVVGGSIPRRMWKCNME
Danio rerio (Zebrafish)     292-YVVGGSIPRRMWKCNMH
```

Supplementary Fig. S1 Sequence conservation analysis for KBTBD4 at the recurrent medulloblastoma mutation hotspots.

A

Interactors	KBTBD4 R313PRR			KBTBD4 P311PP		
	#Unique/Total peptide	Seq coverage %	emPAI	#Unique/Total peptide	Seq coverage %	emPAI
CoREST1	15/21	71	3.9	9/10	24	1.21
CoREST2	6/6	23	0.63			
CoREST3	13/14	48	2.01	4/5	7	0.4

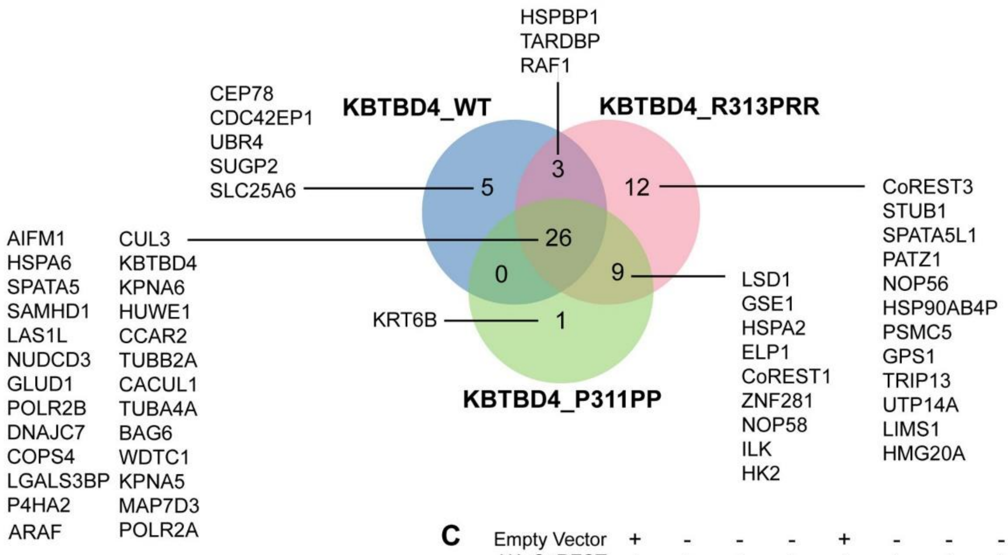

C

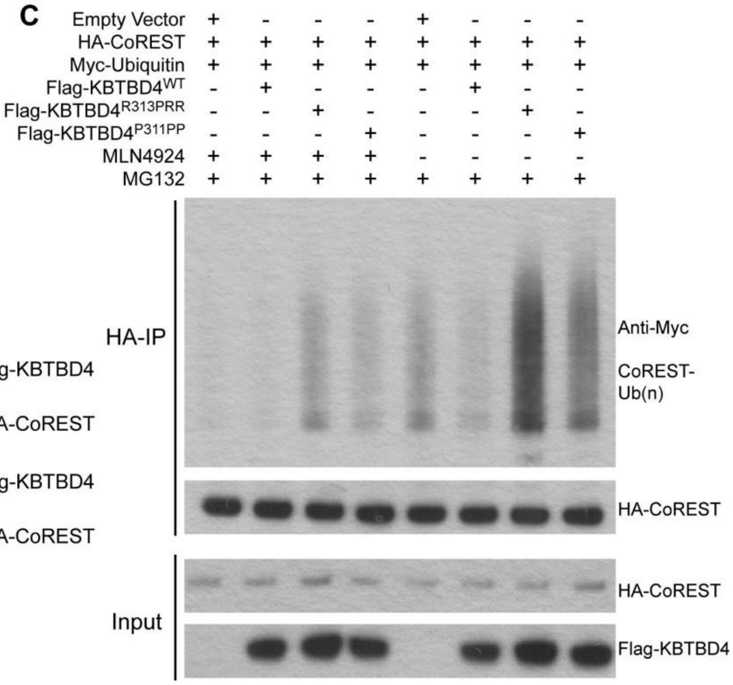

B

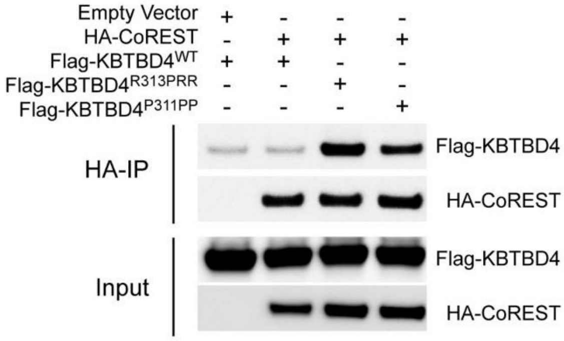

Supplementary Fig. S2 Differential interactions of KBTBD4^{WT}, KBTBD4^{R313PRR} and KBTBD4^{P311PP}. A, Venn diagram of KBTBD4 interactomes identified by LC-MS/MS (showing hits with number of significant peptides >7). The hits in each set are listed**. B,** HA-CoREST and Flag-KBTBD4 variants were co-transfected into HEK293T cells as indicated (+). Cell extracts were immunoprecipitated (IP) with anti-HA resin and immunoblotted as indicated. **C,** HEK293T cells were co-transfected with MYC-tagged ubiquitin and HA-CoREST along with

Flag-KBTBD4^{WT}, Flag-KBTBD4^{R313PRR}, Flag-KBTBD4^{P311PP} as indicated (+). HA-CoREST was IP with anti-HA resin under denaturing conditions and immunoblotted. Cells were treated with 5 µM MG132 and 200 nM MLN4924 as indicated for 4 hours before harvesting. The poly-ubiquitylated CoREST (indicated by brackets) was detected by immunoblot with anti-Myc antibody.

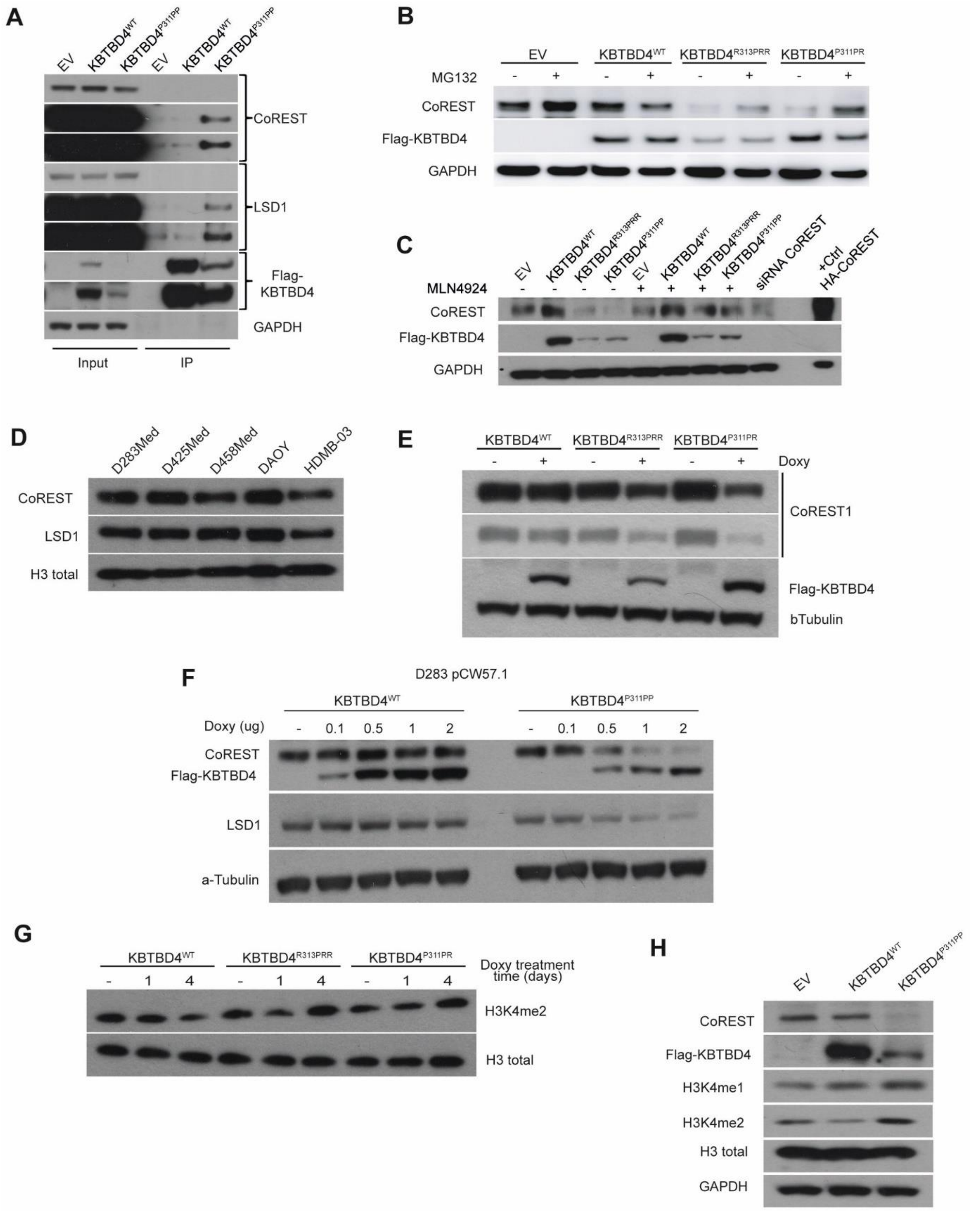

Supplementary Fig. S3 KBTBD4 mutants expressed in different medulloblastoma cell lines drive CoREST degradation and alterations of epigenetic markers. A, DAOY cells were infected with PGK vector expressing an empty vector (EV), Flag-KBTBD4^{WT} or Flag-KBTBD4^{P311PP}. After treatment with MLN4924 for 4 hrs, cells were lysed and extracts immunoprecipitated with anti-Flag resin and immunoblotted as indicated. **B** and **C,** DAOY cells constitutively expressing

an empty vector (EV), Flag-KBTBD4^{WT}, Flag-KBTBD4^{R313PRR} or Flag-KBTBD4^{P311PP} were treated with **(B)** MG132 or **(C)** MLN4924 as indicated (+). Cells were lysed, protein extracts were separated by SDS-PAGE and immunoblotted as indicated. In **C**, a siRNA targeting CoREST and lysate from HEK293T cells expressing HA-CoREST were used as controls to detect CoREST. **D,** Endogenous CoREST and LSD1 protein levels in D283Med, D425Med, D458Med, DAOY and HDMB-03 medulloblastoma cell lines. **E,** D425Med with inducible expression of Flag-KBTBD4^{WT}, Flag-KBTBD4 ^{R313PRR} or Flag-KBTBD4^{P311PP} were treated with doxycycline (1 µg/mL) for 2 days. Proteins were extracted and separated by SDS-PAGE. Samples were immunoblotted as indicated. **F,** D283Med cells with inducible expression of Flag-KBTBD4^{WT} or Flag-KBTBD4^{P311PP} were treated with doxycycline at the indicated concentration for 2 days. Proteins were extracted and separated by SDS-PAGE. Samples were immunoblotted as indicated. **G,** D283Med with inducible expression of Flag-KBTBD4^{WT}, Flag-KBTBD4^{R313PRR} or Flag-KBTBD4^{P311PP} were treated with doxycycline (1 µg/ml) for the indicated days. Chromatin bound proteins were extracted and separated by SDS-PAGE. Samples were immunoblotted as indicated. **H**, DAOY cells with constitutive expression of Flag-KBTBD4^{WT}, Flag-KBTBD4^{R313PRR} or Flag-KBTBD4^{P311PP} were lysed and chromatin bound proteins were extracted and separated by SDS-PAGE. Samples were immunoblotted as indicated.

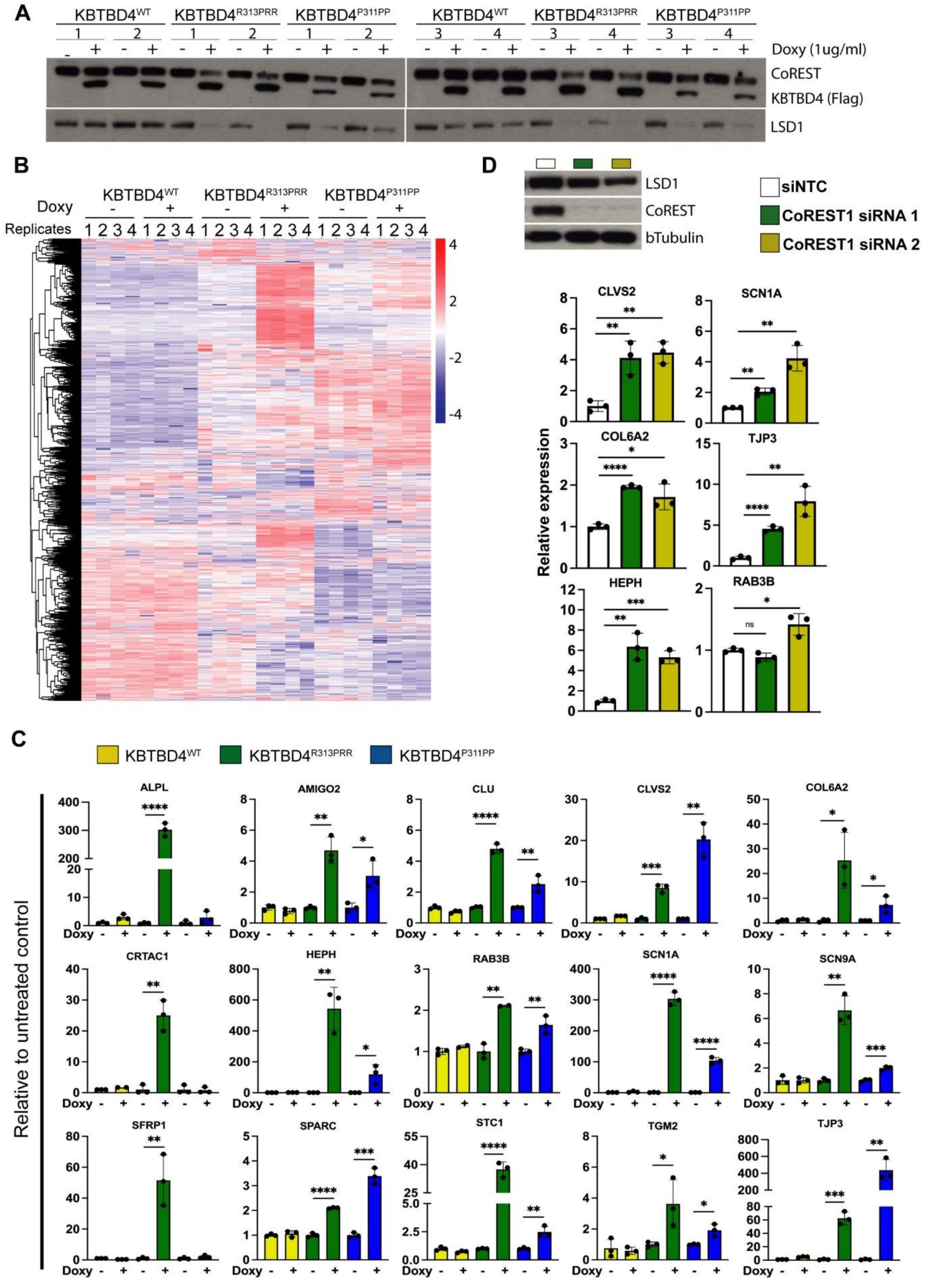

Supplementary Fig. S4 Expression of KBTBD4 mutants promotes transcriptional changes, inducing accumulation of CoREST targets and ES-like signature genes. A, Samples analysed

by RNA-seq (Fig. 3A) were also analysed for protein levels. D283Med cells with inducible KBTBD4^{WT}, KBTBD4^{R313PRR} or KBTBD4^{P311PP} were treated with +/- doxycycline (1 µg/mL) for 4 days as indicated. Proteins were extracted, separated by SDS-PAGE and immunoblotted in respective replicates (1, 2, 3 and 4). Flag-tagged KBTBD4, CoREST1 and LSD1 protein levels are indicated. **B,** Heatmap clustering analysis from D283Med cells expressing Flag-KBTBD4^{WT}, Flag-KBTBD4^{R313PRR} or Flag-KBTBD4^{P311PP} after treatment with doxycycline (1 µg/mL) for four days and same cells left untreated. Samples were run in quadruplicates (1, 2, 3 and 4). Transcripts with *P*-adjust < 0.05 were described as differentially expressed between any two groups, which were profiled as differentially expressed transcripts. **C,** qRTPCR in D425Med cells expressing Flag-KBTBD4^{WT}(yellow), Flag-KBTBD4^{R313PRR}(green) or Flag-KBTBD4^{P311PP}(blue) after doxycycline induction as indicated (+). **D**, qRTPCR in D425Med cells after depletion of CoREST1 by two siRNA. In the top left panel, knockdown of CoREST was validated by immunoblot**.** Bar graphs, qPCR quantification. siNTC - non-target control. *P*-values (**P* < 0.05, ***P* < 0.005, ****P* < 0.0005 and *****P* < 0.00005) were calculated by paired and two-tailed *t*-test.

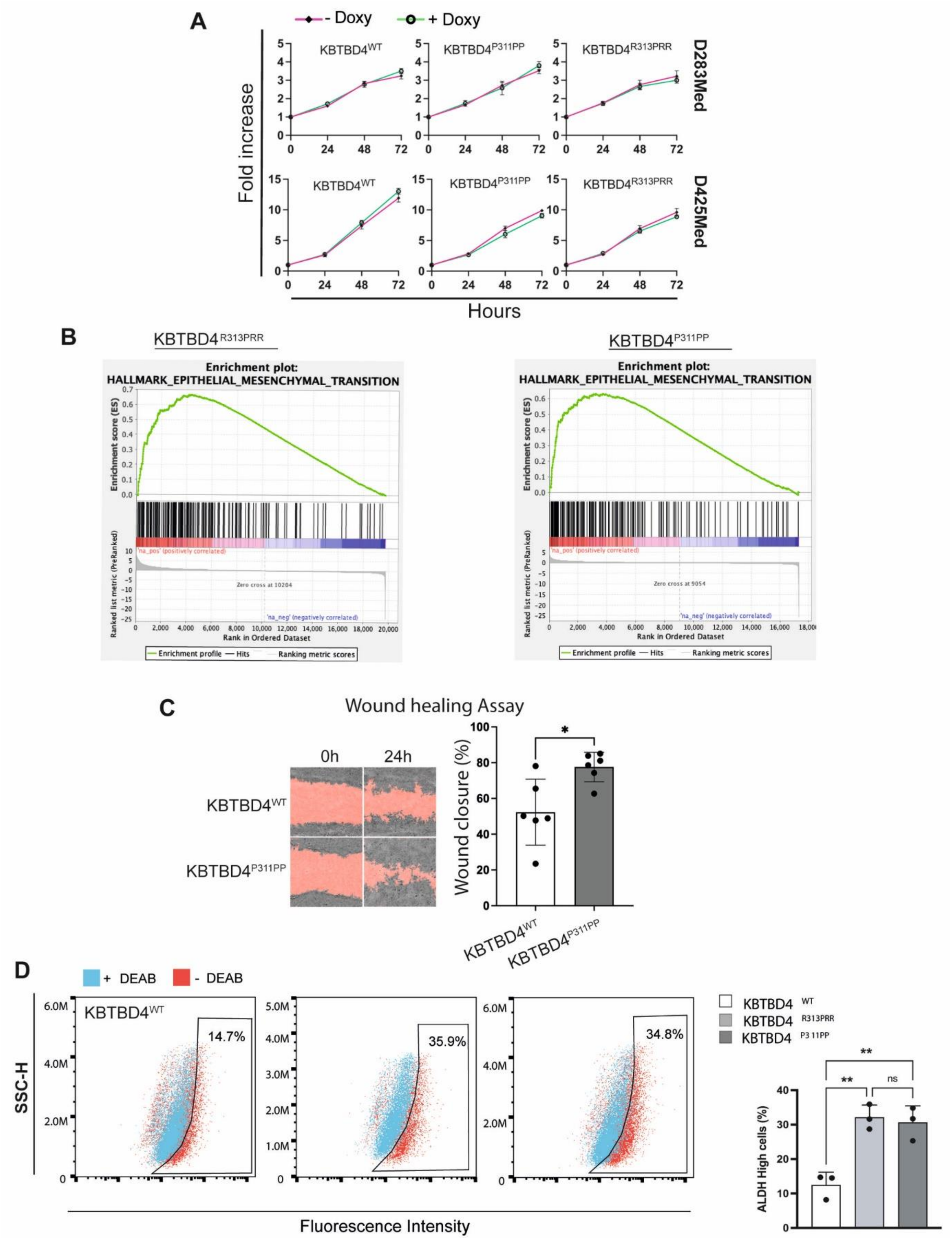

Supplementary Fig. S5 KBTBD4 mutants drive pro-tumorigenic transcriptional programmes and enhance stemness of medulloblastoma cancer cells. A, Measurement of cell growth over 4 days in D283Med and D425Med cells expressing KBTBD4^{WT}, KBTBD4^{R313PRR} or KBTBD4^{P311PP}

after doxycycline addition (1 μg/mL). **B**, Epithelial to mesenchymal transition enrichment plot from Gene Set Enrichment Analysis of RNAseq upregulated genes (*P*-adj <0.05 and log2foldchange >2) between KBTBD4^{R313PRR} or KBTBD4^{P311PP} treated vs untreated samples. **C**, (*Left panel*) Representative images from a wound healing assay of DAOY cells expressing Flag-KBTBD4^{WT} or Flag-KBTBD4^{P311PP}. The wound area (devoted of cells) is shown in orange while cells are shown in grey. (*Right panel*) Quantification of wound closure efficiency as calculated by the percentage of wound area closed at the end point (24h). **D**, Aldehyde dehydrogenase (ALDH) activity, measured as percentage of ALDH positive cells, of D283Med cells expressing KBTBD4^{WT}, KBTBD4^{R313PRR} or KBTBD4^{P311PP}. Panels show representative flow cytometry figures and bar graph with statistics of 3 independent experiments. *P*-values ($*P < 0.05$ and $**P < 0.005$) were calculated by paired and two-tailed *t*-test.
