# Supplementary material for: Disease-associated KBTBD4 mutations in medulloblastoma elicit neomorphic ubiquitylation activity to promote CoREST degradation
Source: Cell Death Differ. 2022 Apr 4;29(10):1955–69. doi: 10.1038/s41418-022-00983-4 (PMC9525703; doi:10.1038/s41418-022-00983-4)

Figure 1D

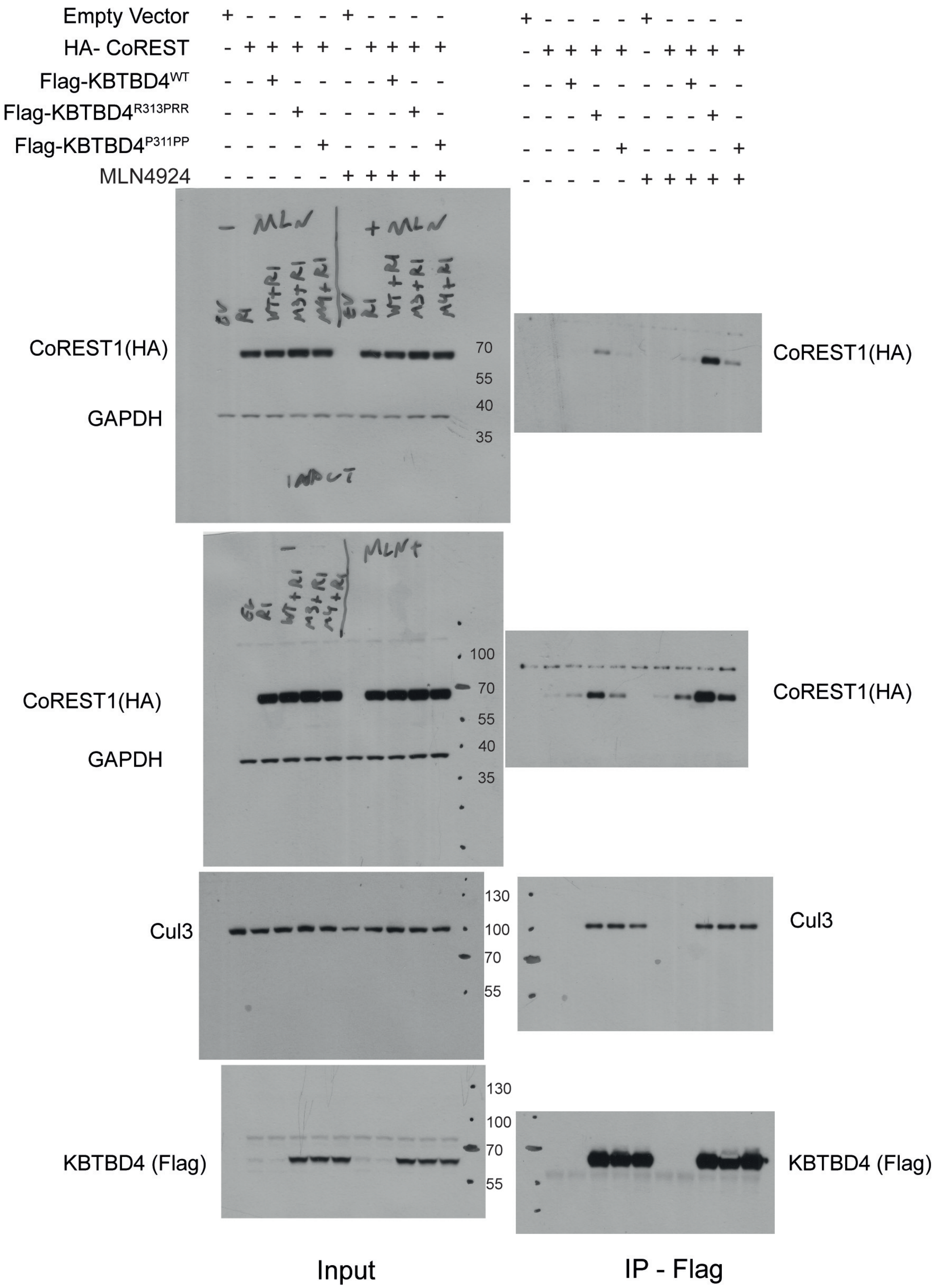

Empty Vector
HA- CoREST
Flag-KBTBD4WT
Flag-KBTBD4R313PRR
Flag-KBTBD4P311PP
MLN4924
CoREST1(HA)
GAPDH
Cul3
KBTBD4 (Flag)
130
100
70
55
40
35
Input
IP - Flag

Figure 1E

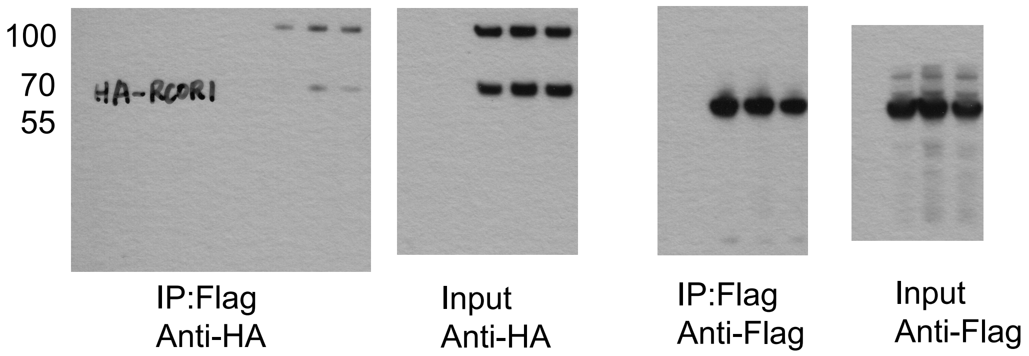

Figure 1F

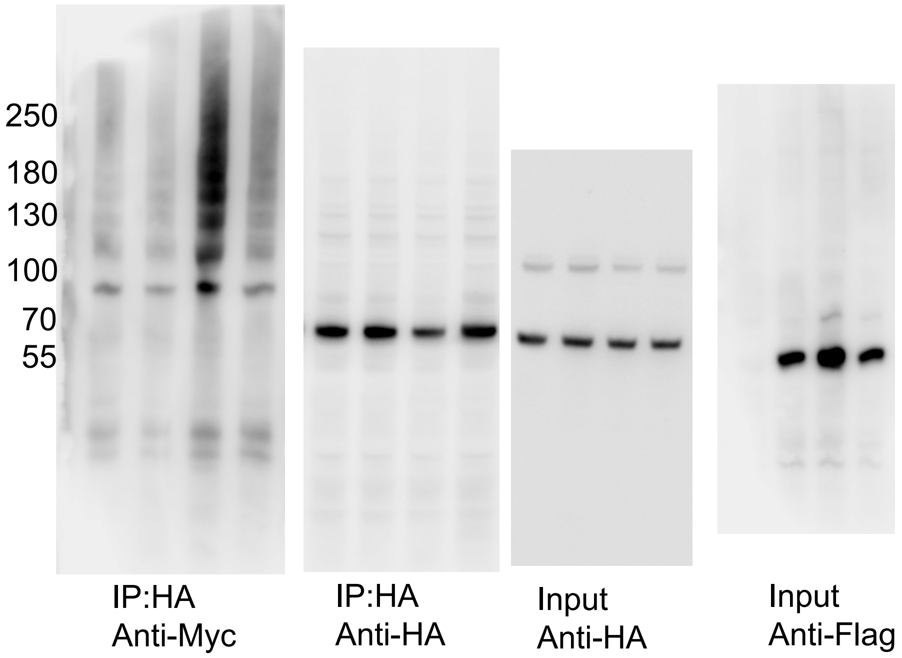

Figure 2A

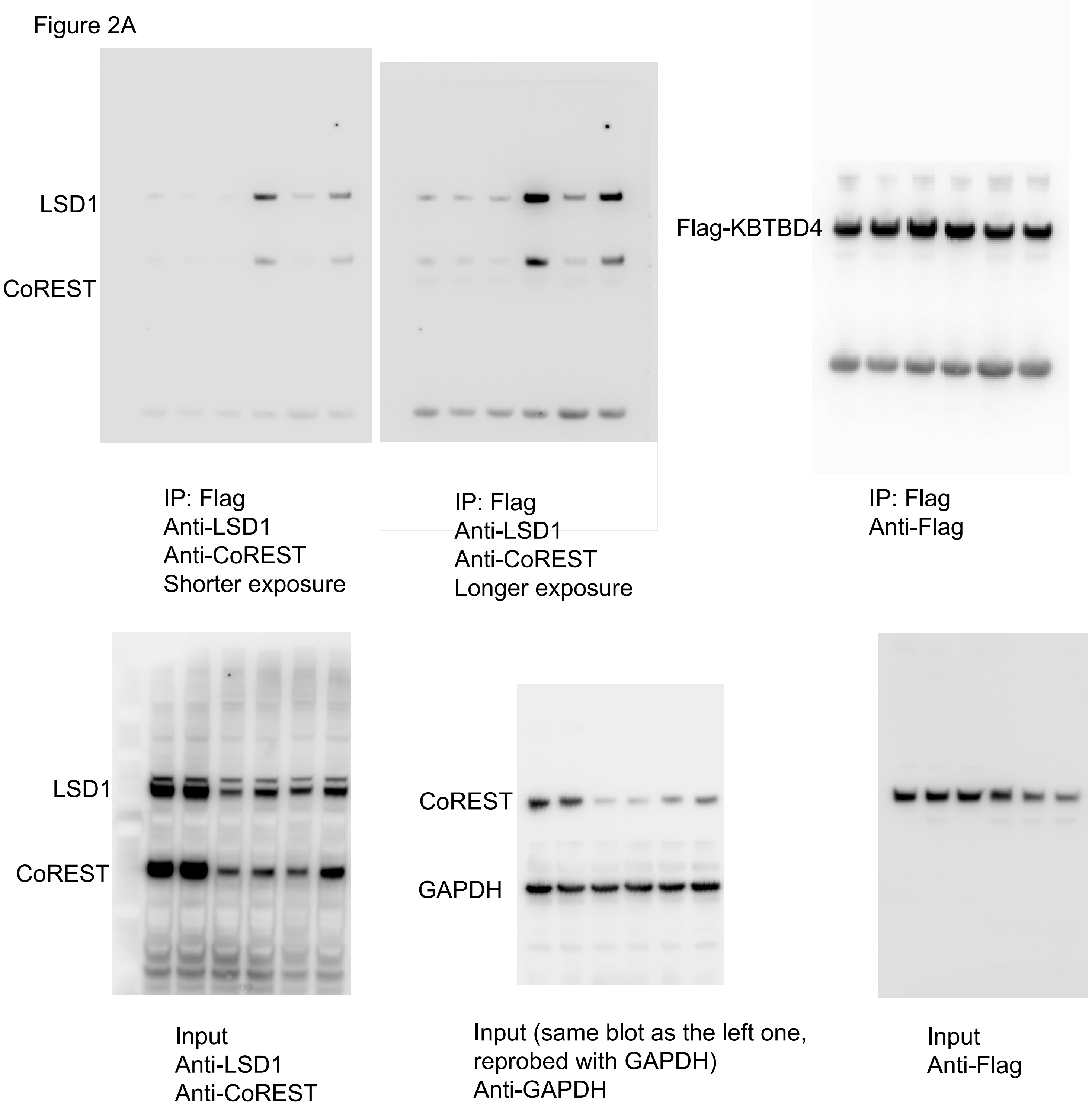

Figure 2B

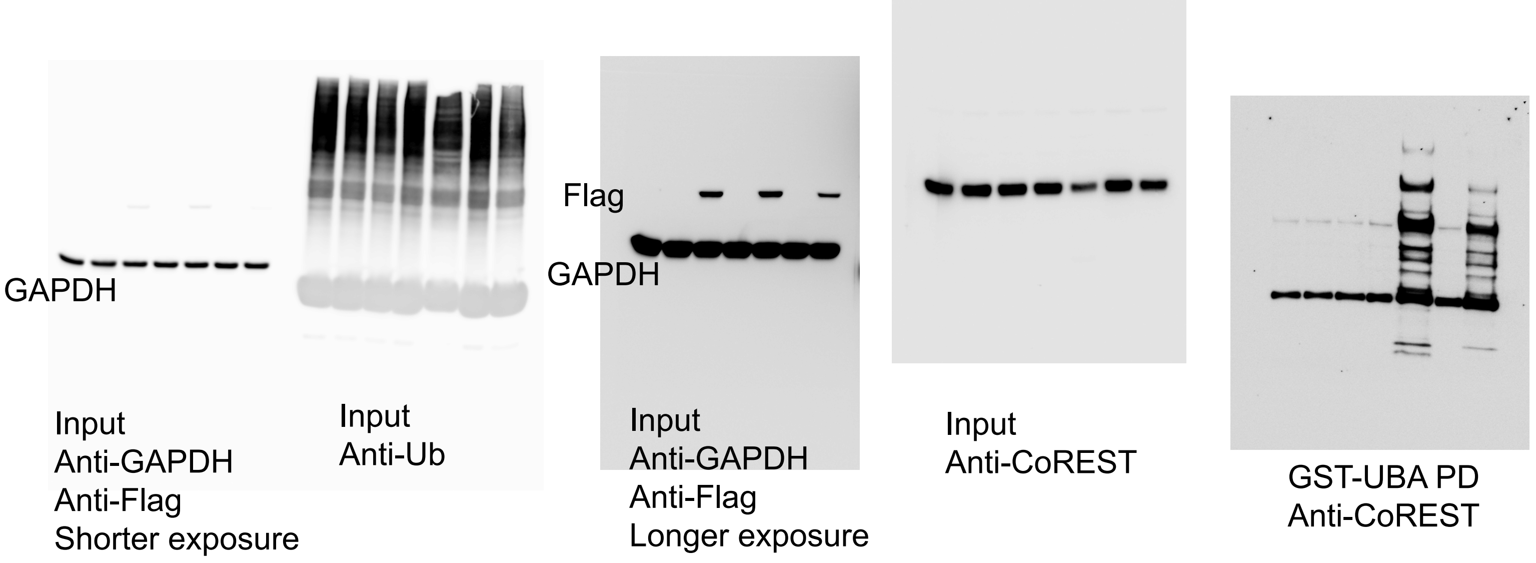

Figure 2C

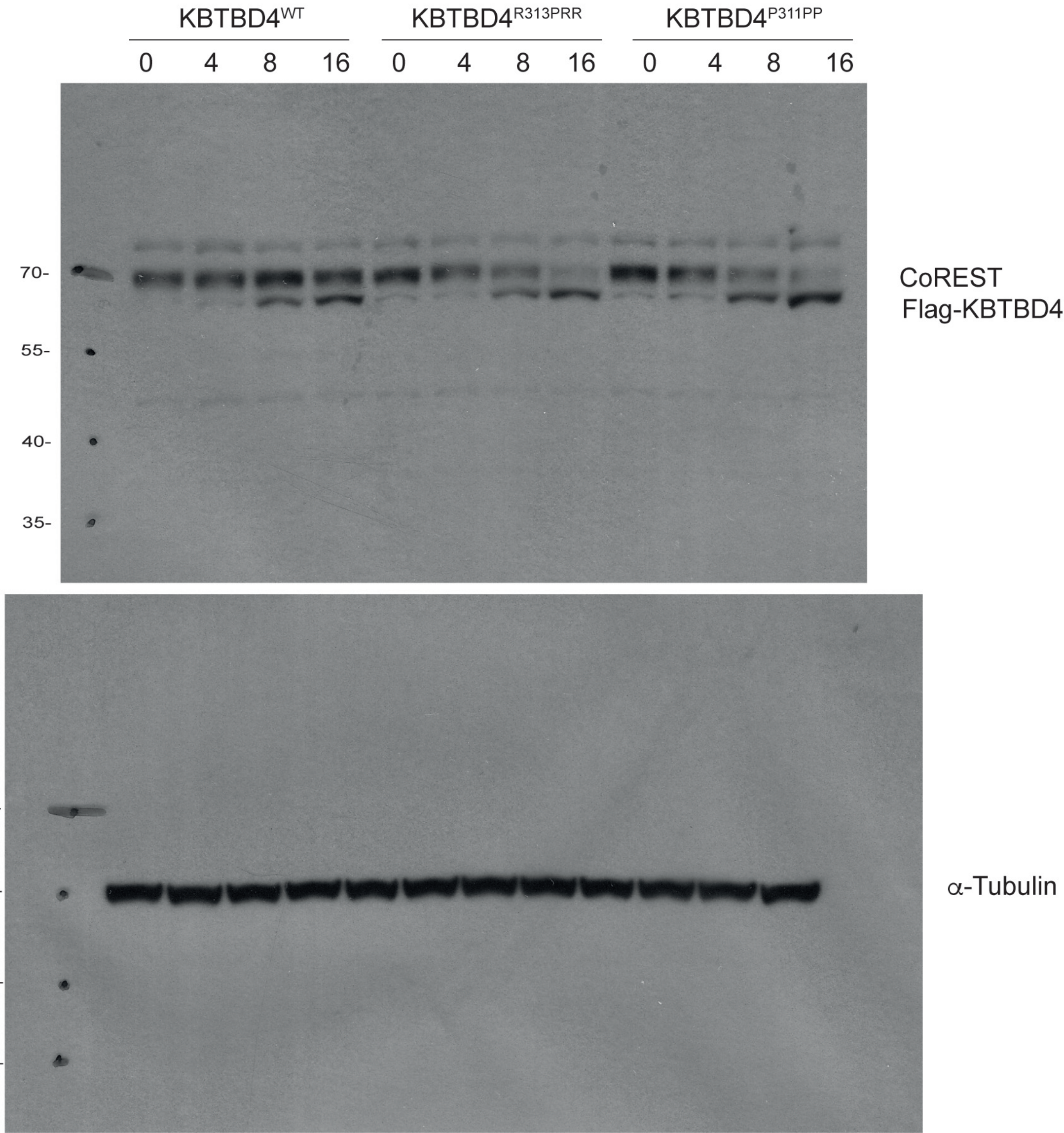
KBTBD4^WT
KBTBD4^R313PRR
KBTBD4^P311PP
0
4
8
16
0
4
8
16
0
4
8
16
70-
55-
40-
35-
CoREST
Flag-KBTBD4
70-
55-
40-
35-
α-Tubulin

Figure S2B

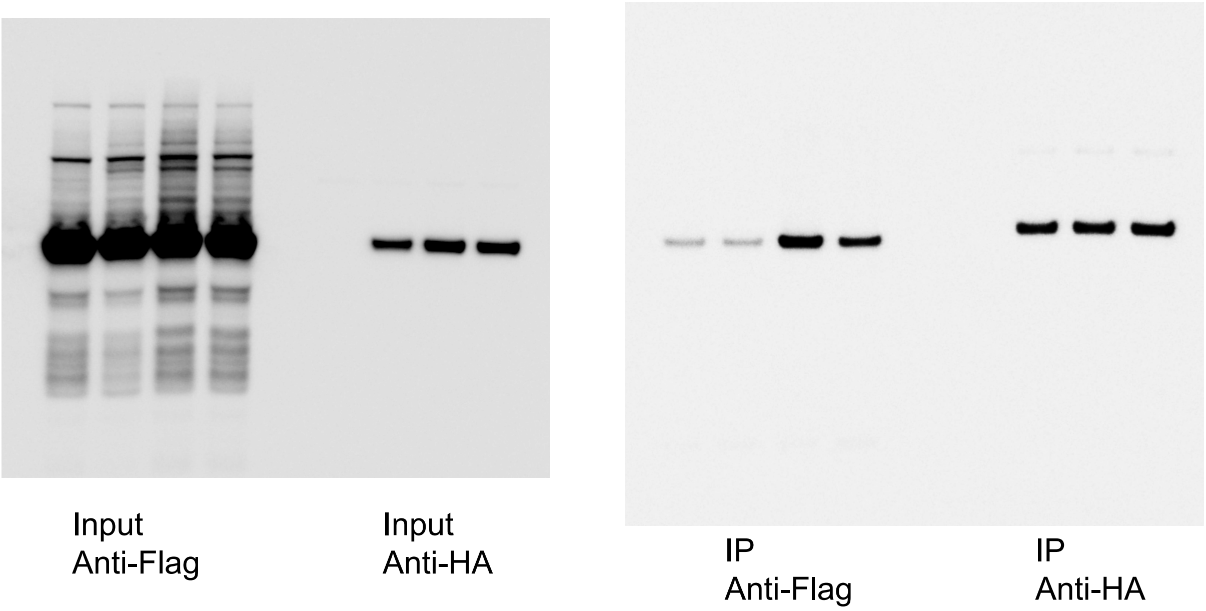

Figure S2C

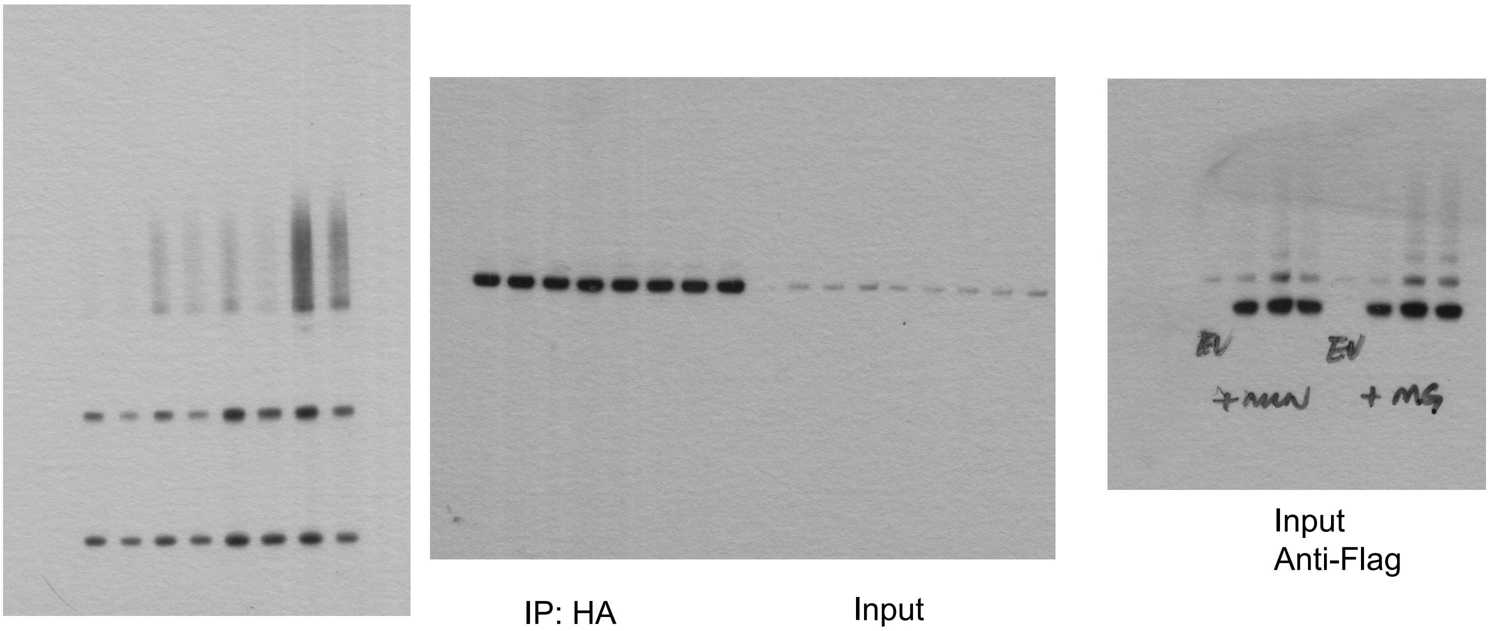

Figure S3A

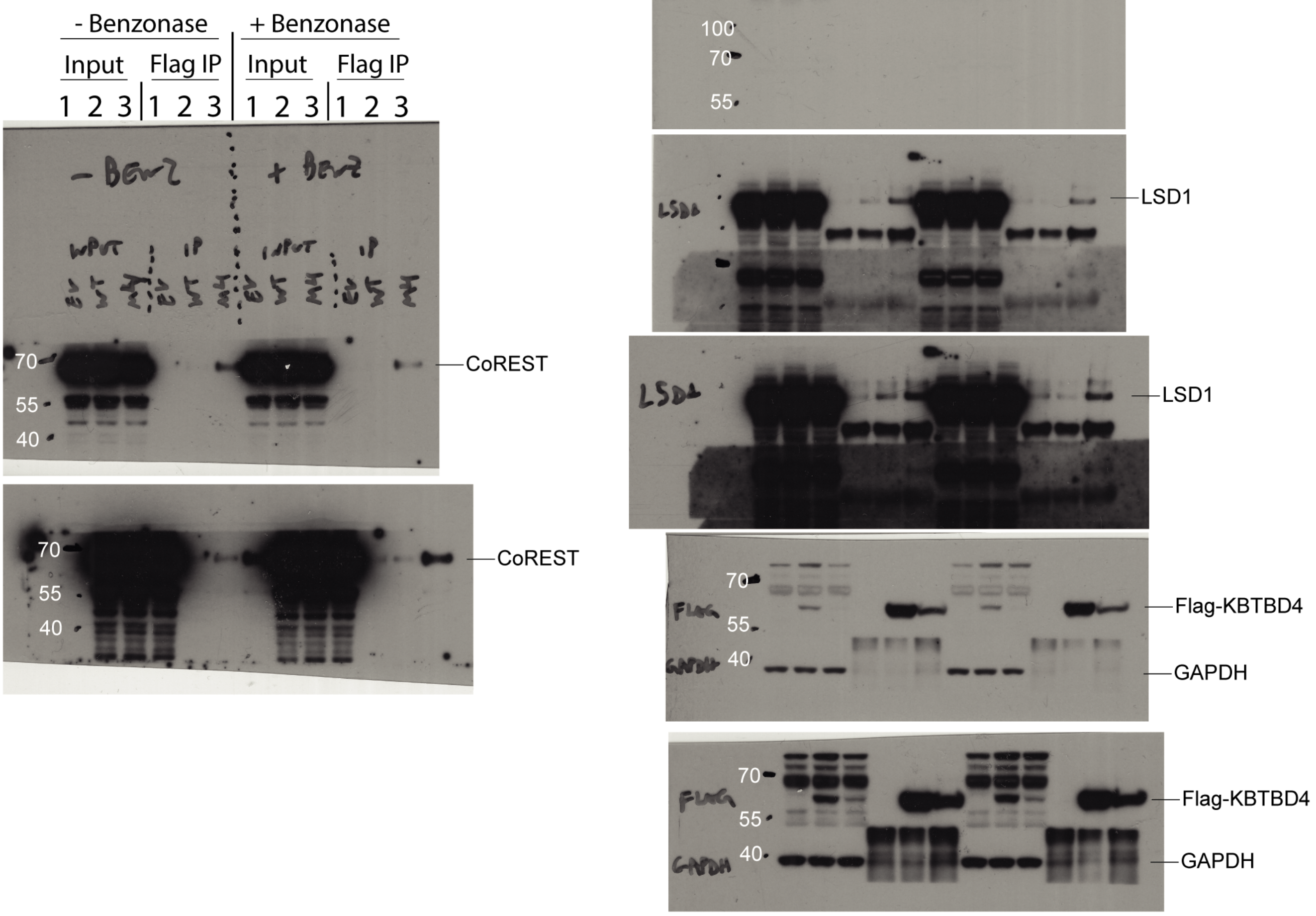

Figure S3C

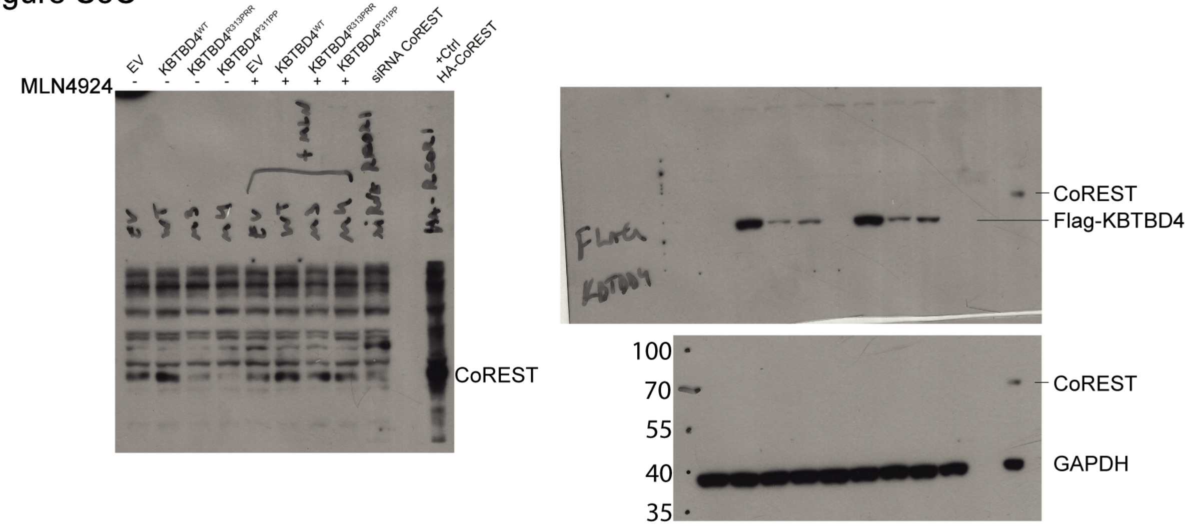

Figure S3D

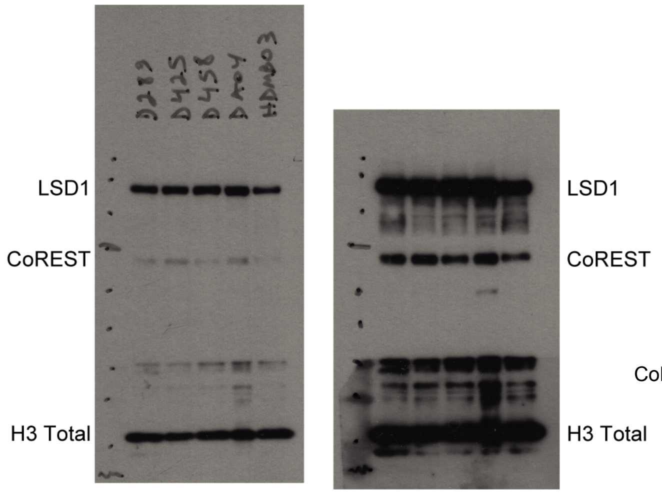

Figure S3B

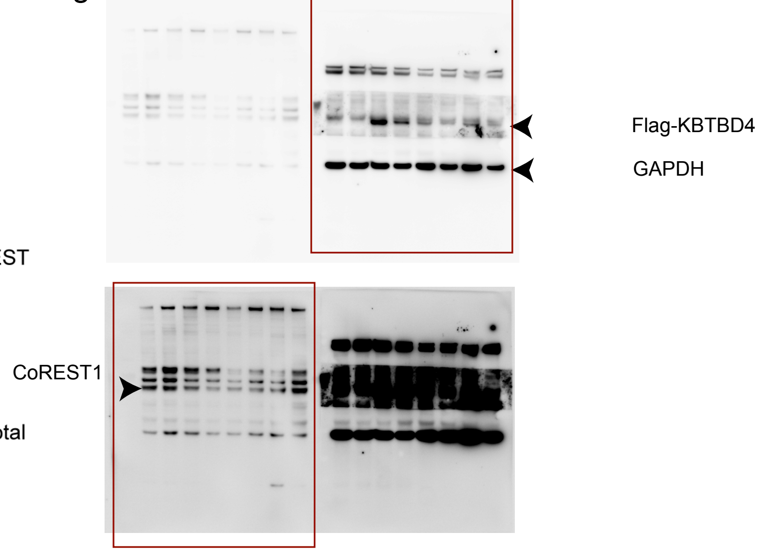

Figure S3E

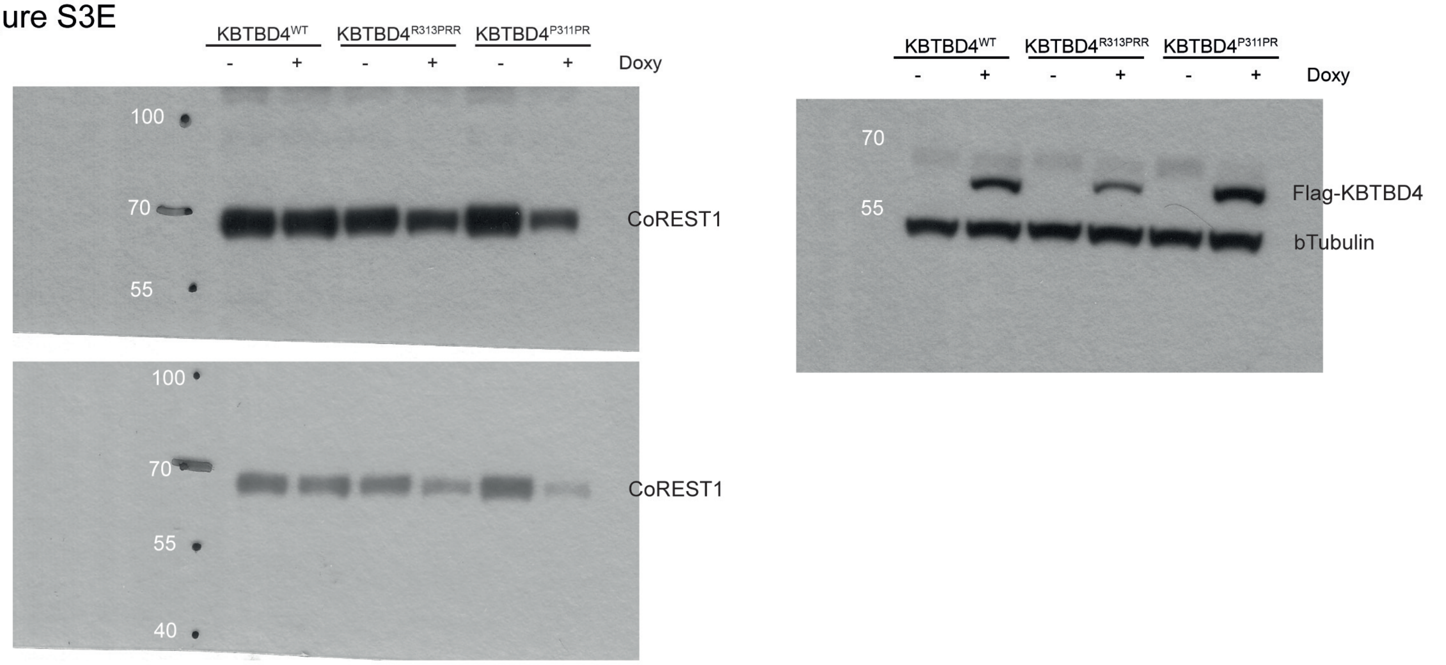

Figure S3F

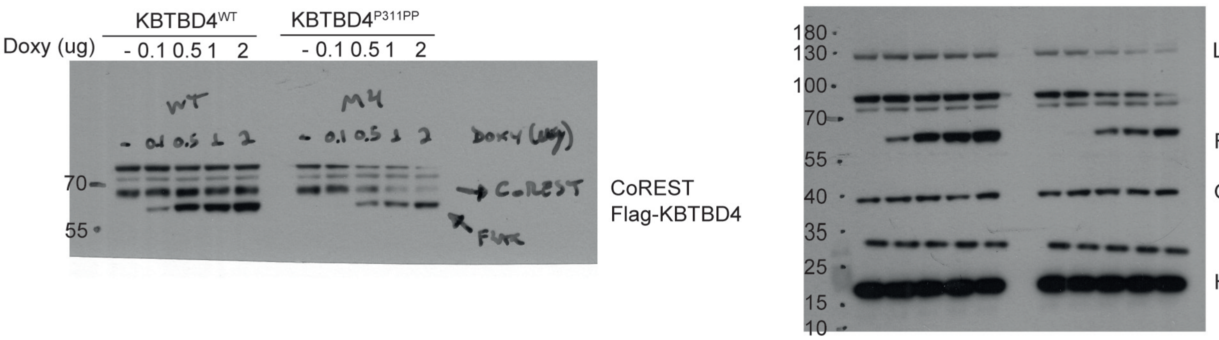

Figure S3G

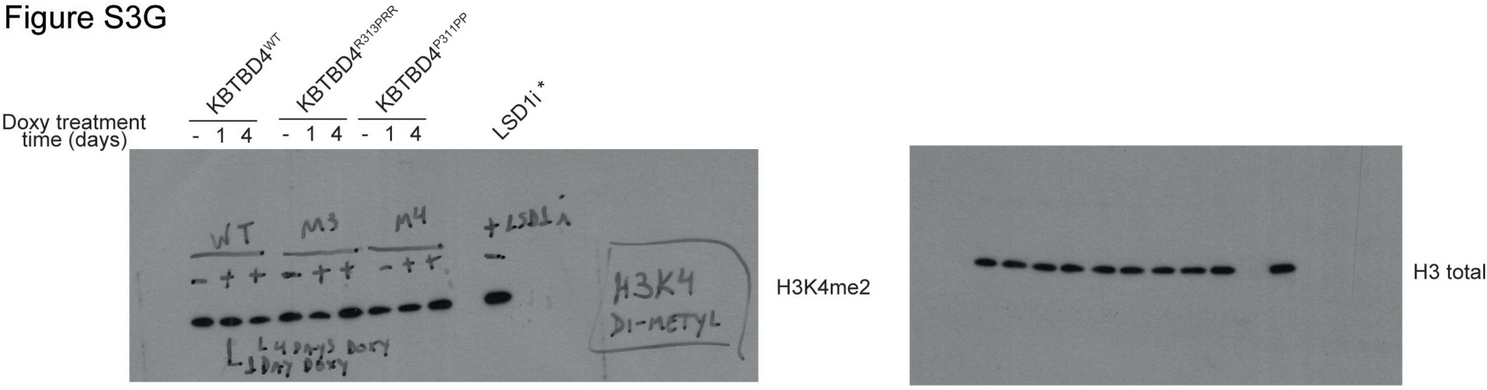

* not shown in this study

Figure S3H

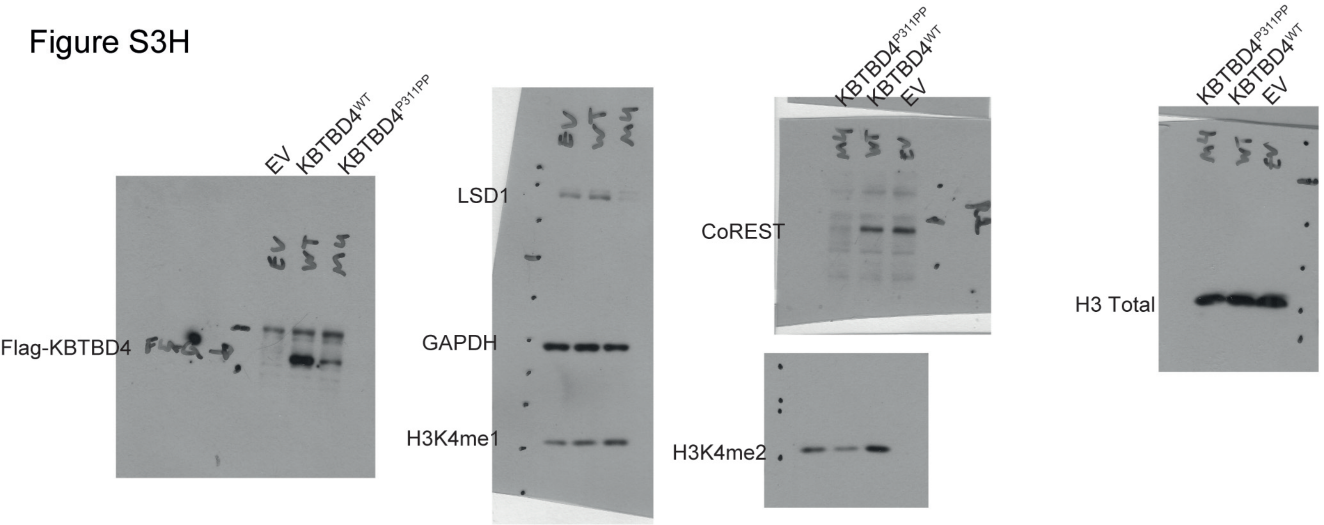

Figure S4A

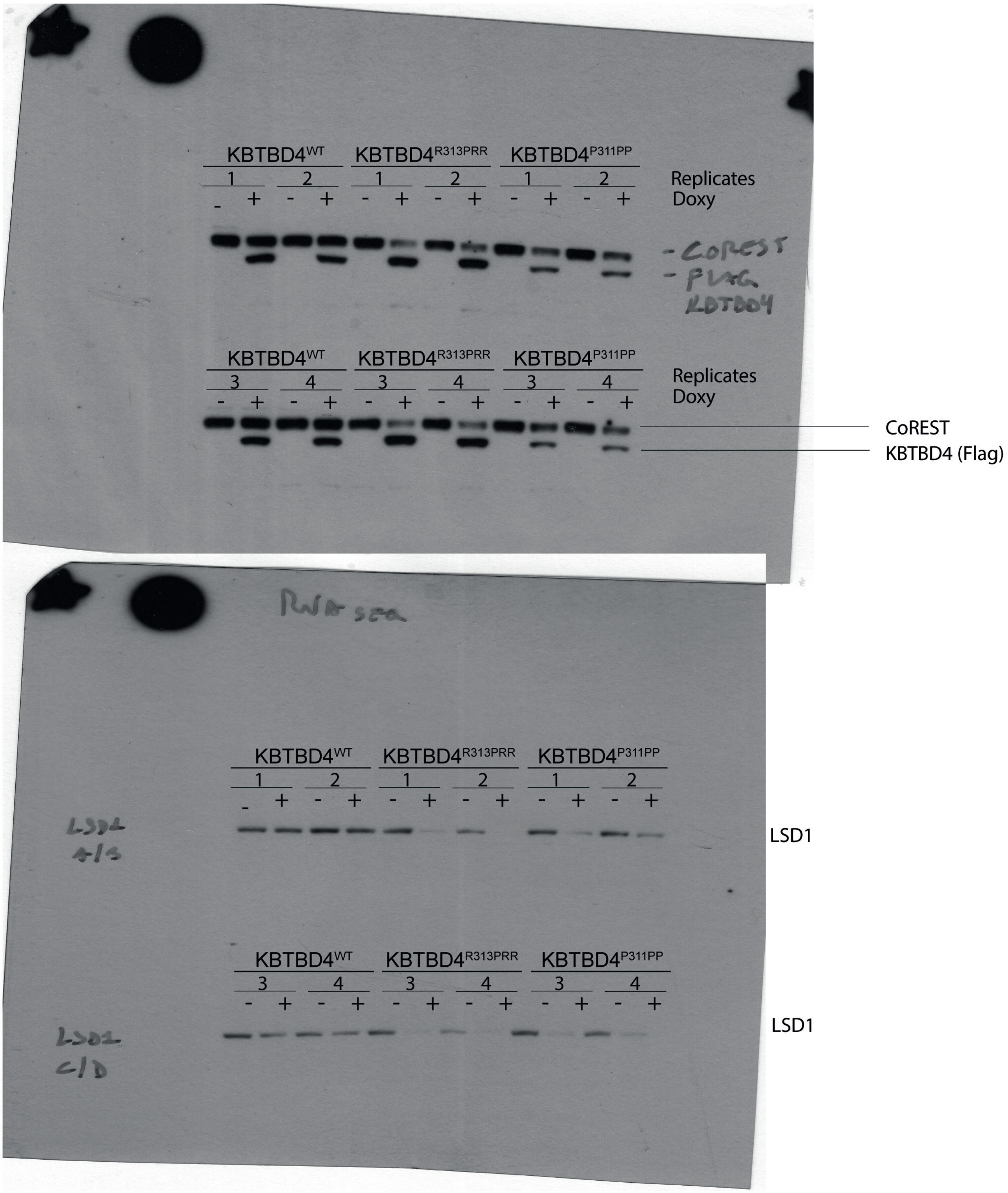

Figure S4D

1 = siNTC

2 = CoREST1 siRNA 1

3 = CoREST1 siRNA 2

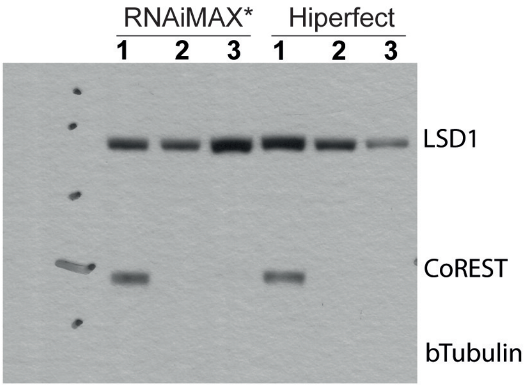

* not shown in this study

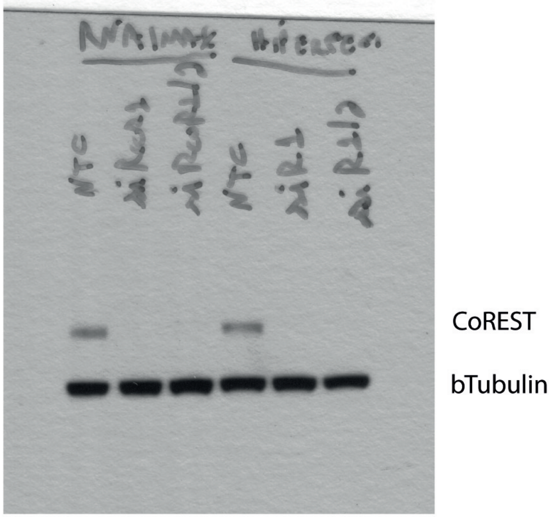

Supplement: Supplementary file 2 — Original Western blot images [file 41418_2022_983_MOESM2_ESM.pdf]
